# The Top 100 Most Cited Articles on Platelet-Rich Plasma Use in Regenerative Medicine—A Bibliometric Analysis—From the ESSKA Orthobiologic Initiative

**DOI:** 10.3390/bioengineering9100580

**Published:** 2022-10-19

**Authors:** Anouck Coulange Zavarro, Laura De Girolamo, Lior Laver, Mikel Sánchez, Thomas Tischer, Giuseppe Filardo, Florence Sabatier, Jérémy Magalon

**Affiliations:** 1 Cell Therapy Department, Hôpital de la Conception, Assistance Publique des Hôpitaux de Marseille (AP-HM), INSERM CIC BT 1409, 13005 Marseille, France; 2 Orthopaedic Biotechnology Laboratory, IRCCS Istituto Ortopedico Galeazzi, 20161 Milan, Italy; 3 Department of Orthopaedics, Hillel Yaffe Medical Center (HYMC), Hadera 38100, Israel; 4 Arthrosport Clinic, Tel-Aviv, Israel; 5 Rappaport Faculty of Medicine, Technion University Hospital, Israel Institute of Technology, Haifa 32000, Israel; 6 Arthroscopic Surgery Unit, Hospital Vithas Vitoria, 01008 Vitoria-Gasteiz, Spain; 7 Advanced Biological Therapy Unit, Hospital Vithas Vitoria, 01008 Vitoria-Gasteiz, Spain; 8 Department of Orthopaedic Surgery, University of Rostock, 18051 Rostock, Germany; 9 Service of Orthopaedics and Traumatology, Department of Surgery, EOC, 6900 Lugano, Switzerland; 10 Faculty of Biomedical Sciences, Università della Svizzera Italiana, 6900 Lugano, Switzerland; 11 Applied and Translational Research Center, IRCCS Istituto Ortopedico Rizzoli, 40136 Bologna, Italy; 12 INSERM, INRA, C2VN, Aix Marseille Univ, 13005 Marseille, France; 13 SAS Remedex, 13008 Marseille, France

**Keywords:** platelet-rich plasma, plasma rich in growth factors, regenerative medicine, bibliometric analysis

## Abstract

Over the past few decades, more and more articles about platelet-rich plasma (PRP) use in regenerative medicine have been published. The aim of this study was to determine which articles have been most influential in this field by identifying and analyzing the characteristics of the 100 most cited articles. Articles on the use of PRP in regenerative medicine were identified via the Thomson ISI Web of Science database. A majority of the articles originated from the USA (36%). The top journal in terms of number of articles was *American Journal of Sports Medicine* (12%). Musculoskeletal system and orthopedics (54%) were the most popular fields of applications. Preclinical studies were the most represented study type, from which only 8 from 46 (17.4%) provided a complete numerical description of the injected product. Analysis showed a time-dependent trend of increasing quality of the clinical studies (*p* = 0.004), although none of them provided a complete biological characterization of the injected PRP. This study demonstrated that the use of PRP in regenerative medicine is a growing and popular area of research, mainly focused on orthopedic applications. Studies on PRP-derived exosomes, biological characterization, and correlation with clinical results might be areas of future trends.

## 1. Introduction

Regenerative medicine is the branch of medicine that develops methods to regrow, repair, or replace diseased and injured cells, tissues, or organs. This field has shown intense expanse and evolution in the past four decades [1]. These variable regenerative medicine therapies can be sorted according to their complexity of manufacturing process: on one hand, gene therapy [2], stem cells [3], or bioprinted tissues, which present more complex procedural steps [2,3,4], and on the other hand, “point of care” autologous therapies involving adipose tissue (nanofat [5], stromal vascular fraction [6]), bone marrow concentrate [7], and platelet-rich plasma (PRP) [8], which are easier to obtain and save time [6,7,8] to manufacture. This last one has emerged as a popular and promising treatment modality in regenerative medicine. PRP is an autologous biological product containing numerous bioactive proteins such as growth factors (IGF, TGF, VEGF, PDGF, etc.), cytokines, and chemokines, which have the potential to play therapeutic roles in a variety of treatments, including musculoskeletal conditions, gynecology, urology, plastic surgery, ophthalmology, and dermatology, while its production remains easy, fast, and quite cheap to set up [9,10,11]. Indeed, PRP is simply obtained by the centrifugation of anticoagulated whole blood, allowing separation of plasma and platelets and having the lowest densities compared to red blood cells (RBCs) and leukocytes. Different authors have classified PRP based on the presence or not of leukocytes, defining leukocytes as rich in PRP if their concentration is higher in PRP compared to whole blood or pure PRP in the opposite case [12,13,14,15]. This differs from Platelet-rich Fibrin (PRF), corresponding to a fibrin matrix whose structure traps platelets and their cytokines, which is obtained after centrifugation of non-anticoagulated blood [16]. Historically, PRP therapy was first described as a surgical adjuvant. Robert Marx is a maxillofacial surgeon who is considered the pioneer of this therapy and defined PRP as ‘‘a volume of autologous plasma that has a platelet concentration above baseline’’ [17].

Thanks to news media reporting the use of PRP products by elite athletes and celebrities [18], it has become a popular procedure over the last few years. A growing number of manual and mechanical procedures have been developed by manufacturers to produce PRP, with about 50 different kits currently available on the market [19]. Despite the popularity and massive use of PRP in regenerative medicine, medical practices remain heterogeneous, limiting its adoption as a standard of care in therapeutic strategies [20]. Several recommendations [21,22,23,24] and classifications [12,13,14,15,21,25,26] have emerged, encouraging both standardization and characterization of the injected biological products.

Due to the complexity of this field and the heterogeneity in clinical practice, the goal of this article is to identify the 100 most-cited articles published concerning the use of PRP in regenerative medicine that have made key contributions to the field, describing the characteristics of articles, providing a reference for better comprehending the worldwide research, and highlighting potential directions for use in regenerative medicine.

## 2. Materials and Methods

### 2.1. Collection and Allocation of Articles

We searched for all relevant articles on the use of PRP in regenerative medicine by using the Thomson ISI Web of Science database including Web of Science Core Collection, MEDLINE, KCI-Korean Journal Database, Russian Science Citation Index, BIOSIS Citation Index, and SciELO Citation Index. Two researchers (ANONYMIZED) independently identified articles for inclusion to enhance the search sensitivity. The keywords used were “platelet-rich plasma” OR “plasma rich in growth factors (PRGF)” AND “regenerative” AND “medicine”. The search was performed on 9 January 2022, and yielded 9073 results in total, which contained all articles published since 1950. Filtering via “journal articles and reviews”, the search resulted in 8311 documents.

The suitability for inclusion was assessed according to the following criteria: original articles (both preclinical and clinical), meta-analyses, systematic reviews, and classifications, whose subject was mainly the use of PRP or PRGF in regenerative medicine field. Articles concerning the use of platelets concentrate as culture media and the use of blood-derived products different from PRP and PRGF were excluded from the analysis.

Selected articles were ranked by the number of citations, with the exclusion of articles with <110 citations to reduce the workload. We sorted 561 results in descending order according to the total citations. The two independent investigators evaluated the articles for their relevance to the use of PRP within the regenerative medicine field, and articles were selected based on title and abstract. Articles whose subjects were the use of platelet-rich fibrin or the use of platelets for different purposes than regenerative medicine, systematic reviews about regenerative medicine, or when PRP was a minor topic were removed. Any disagreements were discussed between two authors until a consensus was reached. After the review of all included studies, 219 articles remained. These articles were arranged according to number of citations, and the top 100 most cited articles were included in the final analysis.

**Figure 1:**
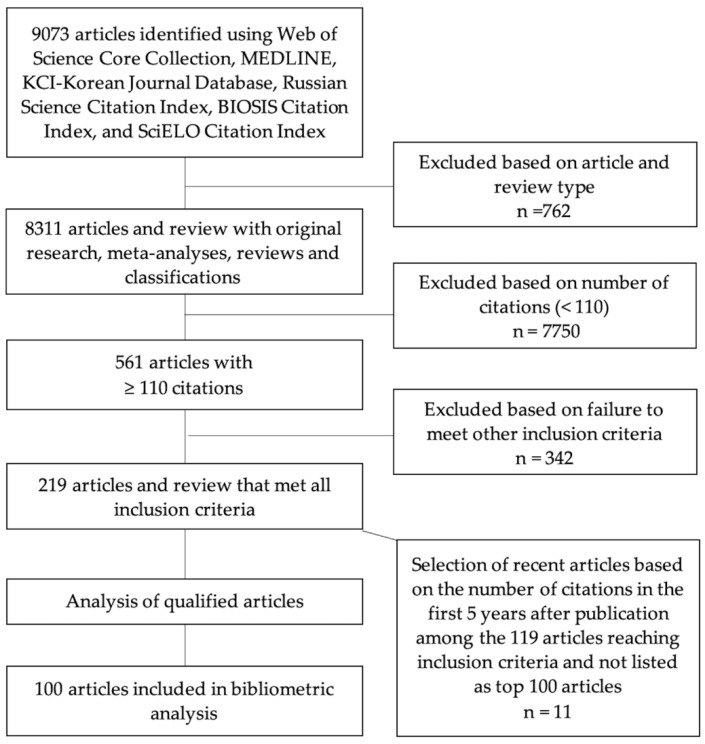
Flowchart illustrating the procedure of allocation of articles.

Since the most recent papers had less time to accumulate citations and to enter the top 100, a further search was performed to overcome this time limit and identify the most promising articles among the latest publications on PRP use in regenerative medicine. As the most recent article from the top 100 was published in 2017, articles published between 2013 and 2017 were analyzed. Among them, a threshold was defined as the minimum number of citations obtained in the first 5 years after their publication. Application of this threshold selected 11 promising articles among the 119 articles reaching inclusion criteria of the study and not yet listed as top 100 articles (Figure 1). The title and abstract were used by the investigators to categorize the themes and type of each article. The themes were (1) biology, (2) cosmetic/plastic surgery, (3) dentistry/maxillofacial surgery, (4) dermatology, (5) musculoskeletal system/orthopedics, and (6) manufacturing. The types were (1) preclinical studies, (2) clinical studies, (3) meta-analyses, (4) reviews, and (5) classifications that were relevant to any aspect of PRP use in regenerative medicine.

### 2.2. Data Extraction

All the selected articles were reviewed independently by the same two authors. The following information was listed for all articles: title, authors name, journal name, year of publication, impact factor of the journal in 2020 (Journal Citation Report or using internal software from an ANONYMIZED hospital when information was not available in the first database), total number of citations of the article, average citations per year (ACY), geographic origin and institutions/corporations of the first author, research theme, and article type. When the first author had more than one affiliation, the department, institution/corporation, and country of origin were reported for each of the affiliations. Subject of investigation was assessed for pre-clinical studies, whereas the following data were collected for clinical studies: indications, number of patients treated in the included studies, mean follow-up, and study design. The level of evidence was first reported from the publishing journal or determined using the Oxford Centre for Evidence-Based Medicine (OCEBM) if it was not [27].

The biological characterization of whole blood and/or PRP was reported for pre-clinical and clinical studies excluding reviews, meta-analyses, and letters. Complete characterization was defined when authors provided biological data on platelets, red blood cells (RBC), and leukocytes concentration both in whole blood (WB) and PRP, and data for at least one growth factor within PRP. Incomplete characterization was defined at least by available data on platelets count in PRP. The absence of biological characterization was reported when none of the above parameters were mentioned in the publication.

An additional assessment of methodological quality of clinical studies was performed using a modified Coleman methodology score (CMS) [28]. Part A was reduced to five parameters for a total of 45 points, and Part B was reduced from 40 to 20 points (Table 1).

### 2.3. Statistical Analysis

The Shapiro–Wilk test was used to test the distribution of individual variables for normality. Data are presented as mean and standard deviation, followed by median and ranges (minimum–maximum), whereas figures are presented using median and ranges. The Kruskal–Wallis test was used to test for differences involving skewed data. The Mann–Kendall trend test was used to test for time-dependent trends. A *p*-value < 0.05 was considered to indicate a statistically significant difference. Statistical analysis was performed via GraphPad Prism, version 9.3.1 (GraphPad Software, San Diego, CA, USA), and XLStat for Excel (Addinsoft, Paris, France/Okayama, Japan/New York, NY, USA).

The citation rank corresponds to the article’s rank based on the total number of citations. The average citation per year (ACY) is the mean number of citations per year since the paper has been published. The mean and standard deviation of total number of citations and ACY do not include decimal value, whereas impact factor includes one decimal value.

## 3. Results

The 100 most cited articles arranged by citation rank are shown in Appendix A, Table A1. The total number of citations was 31,000 (mean ± SD; median (min—max) = 310 ± 232; 249 (173—1879)), including 2562 citations for articles published in the 1990s, 18,267 citations (315 ± 226; 262 (174—1255)) in the 2000s, and 10,171 citations (255 ± 80; 224 (173—515) in the 2010s. Of note, eight articles were cited >500 times.

The Shapiro–Wilk test and the Kolmogorov–Smirnov test both indicated an abnormal distribution of the citation data for all studied parameters (article type, article theme, institutions or corporations, level of evidence).

### 3.1. Characteristics of the Top 10 Most Cited Articles

The top ten most cited articles by number of total citations are listed in Appendix A, Table A2. The mean number of total citations was 853 ± 438 (median (min—max) = 741 (421—1879)). The article classified number ten had 421 citations. The ACY ranged from 20.05 to 75.16. Most of these articles (n = 6) were published between 2000 and 2009.

The first two articles were both published by Robert Marx et al. The article with most overall citations (n = 1879) involved the PRP use in mandibular continuity reconstructions due to oral cancer and was published in *Oral Surgery, Oral Medicine, Oral Pathology, Oral Radiology, and Endodontics Journal* in 1998. This paper reported the results of a randomized controlled trial in which 88 patients received cancellous marrow bone graft with or without PRP.

The second top-cited article (n = 1255) was published in the *Journal of Oral and Maxillofacial Surgery* in 2004. In this review, Robert Marx provided a definition of PRP and collected data concerning its mechanism of action, safety, and therapeutic indications.

The third most cited article (n = 958) was published in Thrombosis and Hemostasis, in 2004 by Eduardo Anitua and colleagues in a review describing the beneficial therapeutic effects of autologous platelets in a wide range of clinical situation.

The article types were clinical studies (n = 3), reviews (n = 4), pre-clinical studies (n = 2), and one classification. Description and conclusions of the top ten most cited articles are presented in Appendix A, Table A2.

### 3.2. Characteristics of the Top 100 Most Cited Articles

**Figure 2:**
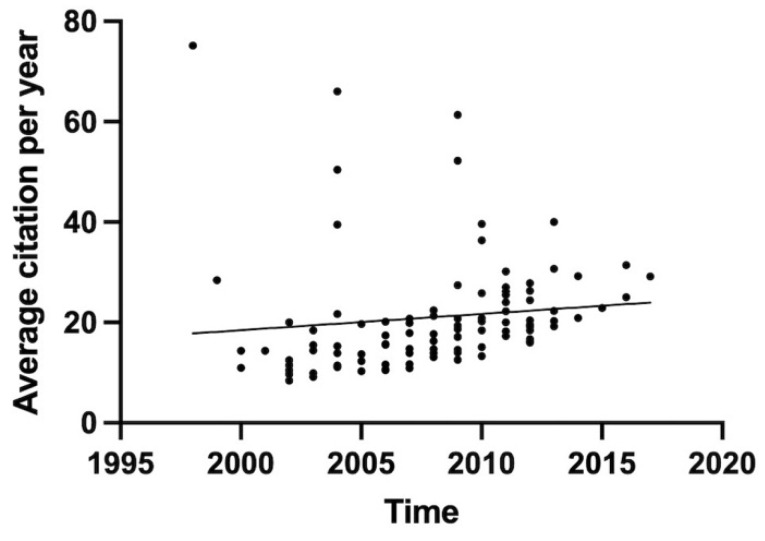
Time-dependent average citation per year trend from 1998 to 2017. The Mann–Kendall trend test showed an increasing trend between citation density and time (*p ≤* 0.0001).

The year of publication ranged from 1998 to 2017, and the majority of the articles were published in the 2000s (58%). The years with the greatest number of articles were 2012 (n = 13) and 2009 (n = 11), followed by 2011 (n = 9). The Mann–Kendall trend test showed an increasing trend between average citation per year and time (*p* < 0.001) (Figure 2).

**Figure 3:**
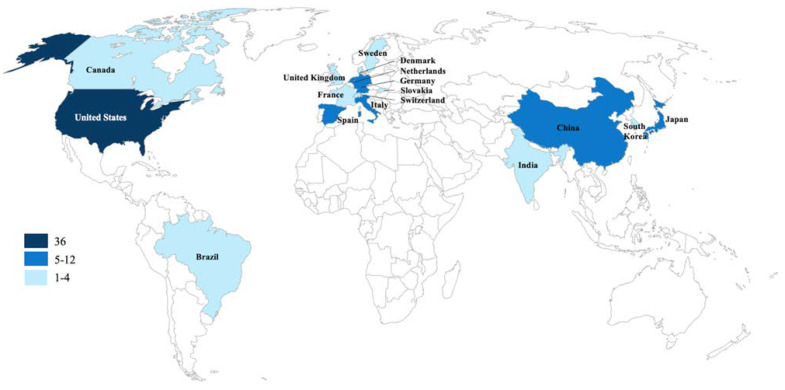
Geographic distribution of the top 100 most cited articles.

**Figure 4:**
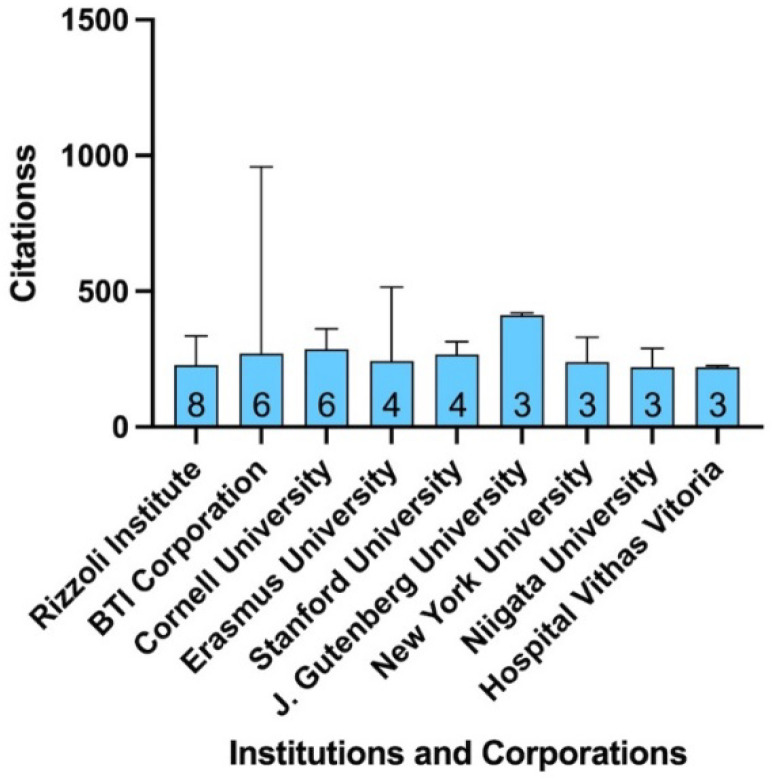
Institutional/corporate distribution of articles. Bar graph showing the median number of citations (and maximum) for the most-cited articles according to the institutional/corporate distribution of articles (number of articles at the bottom of the bar). BTI, Biotechnology Institute; J, Johannes; LA, Los Angeles.

The first author of the top 100 most cited articles originated from 17 different countries. The majority of the articles were from Western Europe (n = 46) and North America (n = 38). The country with the greatest number of published articles was the United States of America (USA) (n = 36) from which first authors were mainly affiliated to universities or corporations located in the states of New York (n = 9; Cornell University: n = 6, University of New York: n = 3) and California (n = 7; Stanford University: n = 4, University of California: n = 2, The Orthobiologic Institute: n = 1) and followed by Massachusetts (n = 4) and Illinois (n = 4). In Europe, the first author affiliation was located in Italy for 13 articles, with the Rizzoli Orthopedic Institute (Bologna, Metropolitan City of Bologna, Italy) (n = 8) and the University of Milano (n = 2) as the most productive. Other productive European countries included Spain (n = 11) and Germany (n = 7), from which Biotechnology Institute located in Vitoria (n = 6) and Johannes Gutenberg University located in Mainz (n = 3) were highly represented, respectively. The first author was affiliated with Japan in eight articles, making this country the most represented in Asia. Geographic origins and affiliations of the first authors from the 100 most cited paper are detailed in Figure 3 and Figure 4. A total of 11 researchers have published more than two publications as first author within the top 100 most cited articles (Table 2).

The most prolific authors were Eduardo Anitua from the Biotechnology Institute Corporation and Giuseppe Filardo from the Rizzoli Orthopedic Institute (n = 7 and n = 4, respectively). Isabel Andia from the Biotechnology Institute Corporation and PierMaria Fornasari from the Rizzoli Orthopedic Institute were the co-authors with the highest number of total publications (n = 10 and n = 8, respectively).

All the top-cited articles were published in 53 journals, led by *AJSM* (n = 12), followed by *Arthroscopy* and *Journal of Orthopedic Research* (n = 6 each), and *Biomaterials* (n = 5). The mean journal impact factor was 5.6 ± 8.1 (median (min—max) = 3.5 (0.4—56.3)) and was higher than 10 for five journals (9%), ranging from 6 to 10 for seven journals (13%). The most represented journals had an impact factor ranging from 3 to 6 (22 journals: 42%) or from 1 to 3 (17 journals, 32%). One journal had an impact factor below 1 (2 %), whereas no impact factor was reported for one journal (2%). The list of journals with a corresponding number of total citations is described in Table 3.

**Figure 5:**
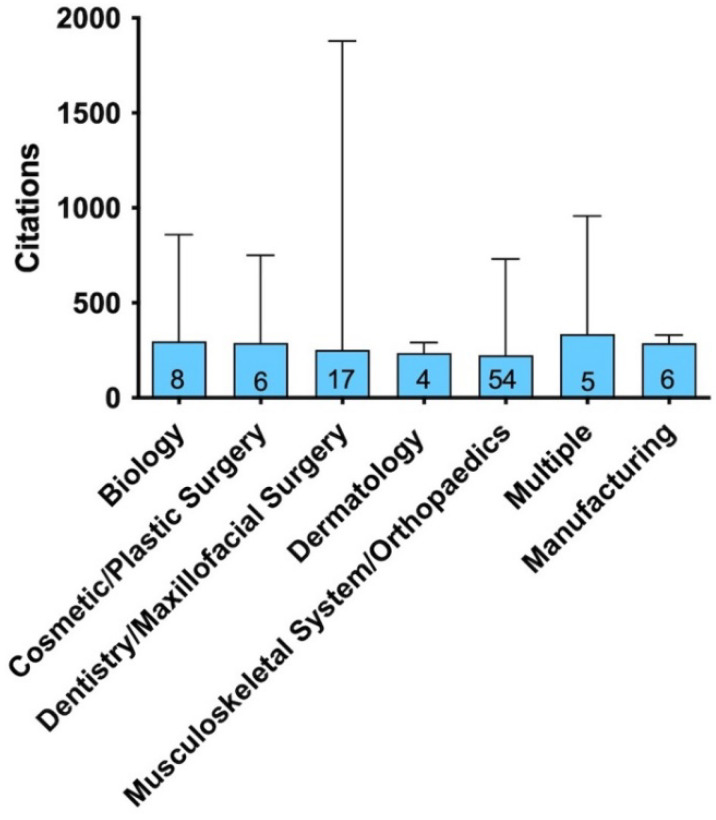
Theme distribution of articles. Bar graph showing the median number of citations (and maximum) for the most-cited articles according to the theme distribution of articles (number of articles at the bottom of the bar).

The top-cited articles were focused on seven disciplines: Musculoskeletal system/Orthopedics (n = 54), Dentistry/Maxillofacial surgery (n = 17), Biology (n = 8), Manufacturing (n = 6), Cosmetic/Plastic surgery (n = 6), Multiple (n = 5), and Dermatology (n = 4) (Figure 5).

**Figure 6:**
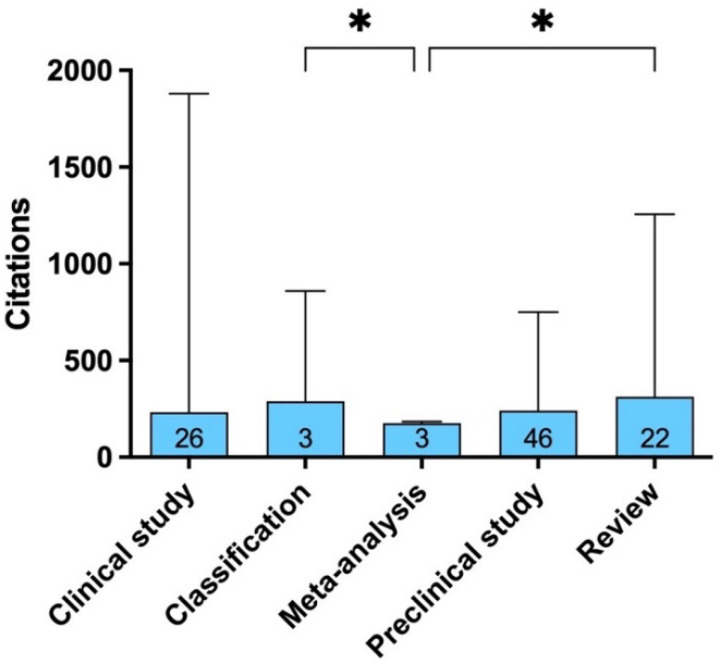
Type distribution of articles. Bar graph showing the median number of citations (and maximum) for the most-cited articles according to the article type distribution of articles (number of articles at the bottom of the bar). * The Kruskal–Wallis test showed significant difference in citations per article among the various types of articles (*p* < 0.05).

The top-cited articles were categorized into “Preclinical Study” (n = 46), “Clinical Study” (n = 26), “Review” (n = 22), and “Classification” and “Meta-analysis” (n = 3 each) (Figure 6).

Preclinical studies include 32 in vitro studies, 11 in vivo studies, and three include both types of experiments (Table 4). The mean impact factor of journals in which clinical studies, preclinical studies, and reviews were published was 5.9 ± 10.4 (4.3 (1.2—56.3)), 4.8 ± 2.6 (3.6 (1.5—12.5)), and 5.8 ± 4.7 (3 (1.0—20.5)), respectively. The Kruskal–Wallis test showed significantly higher citations per article in reviews and classifications compared to meta-analysis (*p* = 0.0051), whereas no significant differences were found based on the themes (*p* = 0.16).

**Figure 7:**
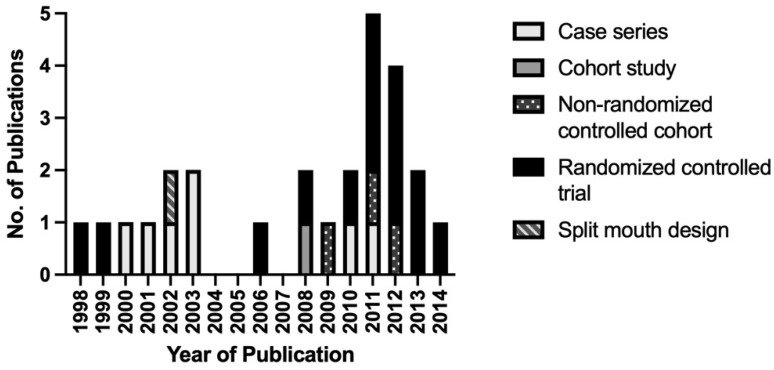
Study design of clinical studies in the top 100 by year of publication.

Evaluation of clinical studies design revealed an increase regarding the proportion of randomized clinical trial within the top 100 cited articles between 2010 and 2014 (Figure 7) that are mainly focused on musculoskeletal diseases (17 from 26 articles, Table 5).

**Figure 8:**
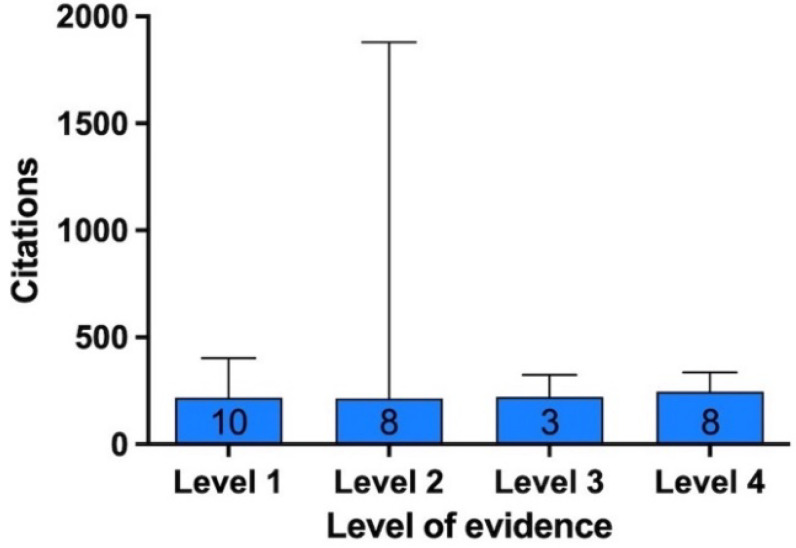
Median and maximum citation per article based on level of evidence. A bar graph showing the median number of citations (and maximum) for the most-cited articles according to level of evidence of articles (number of articles at the bottom of the bar).

Among the clinical studies and meta-analyses, the majority of them were level 1 evidence (n = 10, mean ± SD number of citations; median (min—max) = 240 ± 72; 217 (175—400)), followed by level 2 (n = 8; 514 ± 581; 215 (184—1879)), level 4 (n = 8; 260 ± 49; 246 (204—336)),) and level 3 (n = 3; 249 ± 65; 220 (204—324)). No significant difference regarding the total number of citations among the different levels of evidence of clinical studies and meta-analyses was found (*p* = 0.62) (Figure 8).

**Figure 9:**
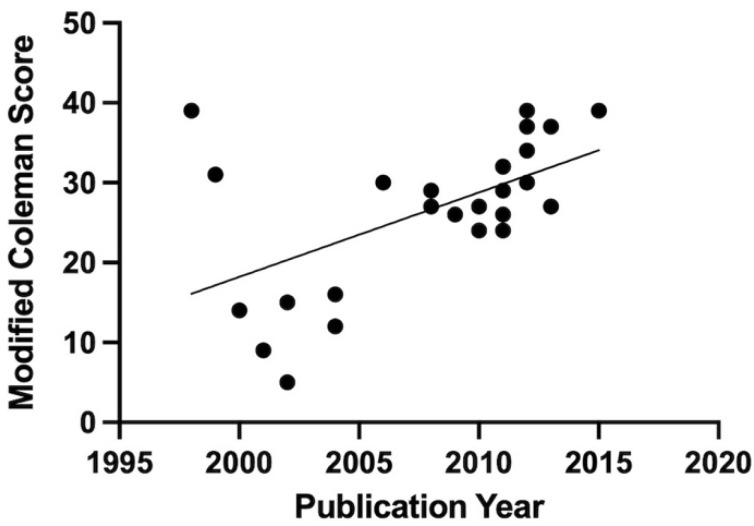
Time-dependent trend of clinical studies modified Coleman methodology score (CMS) from 1998 to 2017. The Mann–Kendall test showed an increasing trend between the modified CMS and time (*p* = 0.004).

Regarding the biological characterization of PRP, only 8 (11%) from the 72 preclinical and clinical studies provided a complete biological characterization of the injected PRP. Interestingly, all these articles were preclinical studies. Overall, 48 publications (67%) provided an incomplete characterization of the PRP injected with systematic reporting of platelets concentration within PRP. Finally, 13 articles (18%) did not report any biological data about the quality of the PRP injected (Supplementary Table S1). The mean modified CMS was 27 ± 9 (median (min—max) = 28 (5—39)) for the 26 clinical studies from the top 100 most-cited articles (Supplementary Table S2). The results showed a time-dependent increasing trend of modified CMS for the top-cited clinical studies when using the Mann–Kendall trend test (*p* = 0.004) (Figure 9).

### 3.3. Most Recent and Promising Articles

The analysis of citations from the articles published from 2013 to 2017 among the top 100 cited articles allowed us to define a threshold of 80 citations in the five years following their publication to define the most promising articles. The second search, conducted on the 119 articles in accordance with inclusion criteria above the top 100 cited articles, allowed us to select a further 11 papers. These articles obtained from 81 to 160 citations in the first five years from publication (105 ± 23 (103 (81—160)).

These articles were preclinical studies (n = 4), clinical studies and meta-analyses (n = 3 each), and one review. Themes of articles were musculoskeletal system/orthopedics (n = 6), dermatology (n = 2), biology, dentistry/maxillofacial surgery, and multiple (n = 1 each). The mean impact factor of journals in which clinical studies, preclinical studies, and meta-analyses were published was 3.6 ± 2.9 (4.2 (0.52—6.2)), 7.9 ± 4.5 (8.9 (2.4—11.6)), and 6.0 ± 2.9 (4.8 (4.0—9.3)), respectively. The impact factor for the review was 13.6 (Table 6).

Modified CMS was 17, 39, and 26 for the most promising recently published clinical studies (Supplementary Table S2).

## 4. Discussion

In 1987, the first bibliometric article was published in *Journal of American Medical Association* (*JAMA)*, launching a tradition for this type of article to be regularly published in scientific literature. Indeed, one way to measure the academic importance of an article is the rate at which the work is quoted or referenced by other authors’ number of citations, which remains a valuable measure of the impact of an article has on a specific topic. Despite its development and clinical use for over thirty years, PRP use has become increasingly popular in the past decade, particularly for treating musculoskeletal injuries.

However, its clinical efficacy remains a matter of debate in the scientific community due to contradictory results that have been published which limit its widespread use. In the context of this innovative and debated treatment, we provide a large bibliometric analysis on PRP use in regenerative medicine by ranking the top 100 articles by number of citations. Article types published until 2007 (n = 42) were represented by preclinical studies (62%), clinical studies (21%), and reviews (17%), whereas from 2008 to 2017 (n = 58) articles were represented by preclinical studies (34%), clinical studies (29%), reviews (26%), meta-analyses, and classifications (5% each) confirming the growing popularity and interest of PRP therapy for clinical use. The results of our bibliometric analysis confirm the position of Robert Marx as a pioneer in the field of PRP use in regenerative medicine (total number of citations of 3134). The American nationality of Marx probably created a wide enthusiasm and investment of North American researchers in this field, as first authors originate from USA make up 36% of the top 100 most cited articles. This aspect may have been reinforced by the important representation of this topic in the American Journal of Sports Medicine (12 from 100 articles).

Browsing through the top 100 list also shows that most articles fall into the use of PRP in the musculoskeletal system (54 from 100 articles), far ahead of the other themes studied.

Surprisingly, preclinical study was the category of most cited articles among the top 100 (n = 46), with a mean impact factor of 4.8 ± 2.6 (median (min—max) = 3.6 (1.5—12.5)). They also represent the only type of study providing a complete biological characterization (n = 8).

Comparatively, the 26 clinical studies listed among the top 100 cited articles reached a mean IF of 5.9 ± 10.4 (4.3 (1.2—56.3)), of which 15 provided at least biological data on platelet count within PRP, whereas 10 did not provide any data related to biological characteristics of the injected product. This lack of characterization of PRP is systematically reported as a weakness in the field [29,30,31,32,33], although three classifications published in 2009, 2012, and 2014, respectively, are listed in the top 100 cited articles. Our top 100 most cited articles show that only 30% of clinical studies are level 1, according to the OCEBM classification. As this classification does not take into account the biological characterization of the PRP, 62.5% of these level 1 studies did not provide any data concerning the product injected. The interest in using the modified CMS was therefore to refine these levels of evidence analyses and to provide more accurate results for clinical studies using PRP. Our analysis showed an overall increasing quality of the clinical studies over time based on the modified CMS. As PRP is a new therapy, and its use remains controversial, it is crucial that future clinical studies strive to provide the highest level of evidence, including both classical applications of the concept of evidence-based medicine [34,35,36] and systematic detailed descriptions of the protocol for obtaining PRP and its related quality controls. In our opinion, researchers should systematically report at least the erythrocyte, leukocyte, and platelet counts of the blood sample and the PRP and, if possible, associated growth factors, which constitute the identity card of the injected product and would make it possible to classify the type of PRP produced. Moreover, this systematic characterization of the PRP would allow establishing correlations between the injected dose of each cellular element and growth factors and the clinical outcome.

The second level of research of this study allowed us to identify promising articles not yet referenced within the top 100, following the methodology recently used by Franceschini et al. [37]. Interestingly, the two first articles were high-level preclinical studies both published in Theranostics (impact factor in 2020: 11.6) on the use of exosomes derived from PRP in diabetic and osteonecrosis rat models, which could be a popular area of research in the future. Indeed, extracellular vesicles (EVs) are the subject of increasing interest due to their ability to transfer biological content between cells. EVs, emitted in the extracellular space, circulate via the different fluids of the organism, and modulate locally or remotely the responses of the cells with which they have interacted [38]. Three meta-analyses (two in musculoskeletal system and orthopedics and one in dermatology) were also listed as promising articles, confirming the growing clinical interest of PRP use in medicine. The presence of four preclinical studies (two in vitro and two with both in vitro–in vivo experiments) in this selection of articles highlights the need for continuing to explore some unrecognized effects of PRP. Topics studied in the three clinical studies (two in musculoskeletal system and orthopedics, one in dentistry and maxillofacial surgery) were comparable to the most popular topics found in the top 100 cited articles.

Although more recent expert recommendations and biological classifications published since 2014 were not listed as promising articles, it is likely they will also reach a high level of citations in the coming years, as three of them already reached more than 50 citations with ACY higher than 10 and were only published in 2015, 2018, and 2020 [15,21,25]. Interestingly, detailed and rigorous biological characterization of whole blood and PRP for each injection performed both in clinical studies or in routine care is part of the American Academy of Orthopedic Surgeons (AAOS), International Society on Thrombosis and Hemostasis (ISTH), American Medical Society for Sports Medicine (AMSSM), and European Society of Sports Traumatology Knee Surgery and Arthroscopy (ESSKA) guidelines. This point is reinforced by the fact that journals of high scientific quality now require the inclusion of a dedicated checklist as a supplemental file and detailing the Minimum Information for Studies Evaluating Biologics in Orthopedics (MIBO), which contains nine items referring to PRP preparation [39,40]. Recommendations are not limited to the musculoskeletal field, as the Academy of International Regenerative Medicine and Surgery Societies (AIRMESS) recently published recommendations about the use of PRP in androgenetic alopecia and wound healing, also encouraging researchers to standardize the use of PRP [41]. It will be interesting to follow the influence of these recent recommendations on the future landscape of publications in these fields, whether will it increase the level of the publications (8 from the 26 clinical studies are level 1 with only 5/54 journals with an impact factor higher than 6) or will participate to a broader diffusion of PRP therapy.

Concerning the 22 reviews listed in the top 100 cited articles, they represent a total of 8410 citations. They mainly describe the mechanisms of action of PRP, detail the role of platelet growth factors, and summarize PRP clinical applications either in a broad way or in a dedicated field. Comparison with a similar bibliometric analysis in a larger field like orthopedic knee research published in 2014 allows us to understand that PRP use remains a small area of research (2640 citations vs. 1879 citations for the first article of the top 100, 47,653 total citations for the top 100 cited articles vs. 31,000, and 29 articles vs. 8 articles with more than 500 citations, respectively) [42]. A recent bibliometric analysis published in 2022 reported the most-cited PRP-related articles in the field of knee osteoarthritis [43], confirming the USA and AJSM as the most productive country and journal on the topic. Interestingly, four of the top five most-cited articles in this bibliometric analysis were present in the 100 we reported in this article. This is consistent with the fact that the majority of the clinical studies on the musculoskeletal system category from our bibliometric analysis were focused on knee osteoarthritis (10/17).

This study has limitations, such as the use of the 2020 impact factor, which is questionable but necessary to classify the different journals. Furthermore, a total number of citations as the first criterion of classification is not appropriate to evaluate the quality of the most recent articles and is not systematically synonymous with quality. However, this parameter remains one of the best and is more often used to evaluate the impact of an article over time, whether or not it brings a positive opinion to the scientific community.

## 5. Conclusions

In conclusion, this bibliometric analysis provides a valuable snapshot of articles that have attracted citations regarding the use of PRP in regenerative medicine. Periodic updates will be necessary to include more recent articles and measure the impact of recent recommendations. The present study revealed that the majority of the top-cited articles were represented by preclinical and clinical studies in the musculoskeletal system and orthopedics. Studies on PRP-derived exosomes, biological characterization, and correlation with clinical results might be areas of future trends.

## Figures and Tables

**Table 1:** Modified Coleman methodology score.

Part A—Only One Score to Be Given for Each of the Sections		
		Score
1. Study Size—number of injected sites	>60	10
	41–60	7
	20–40	4
	<20, not stated	0
2. Mean follow-up (mths)	>24	5
	12–24	2
	<12, not stated or unclear	0
3. Platelet-Rich Plasma characterization	Complete	10
	Incomplete	7
	No data	0
4. Type of study	Randomized controlled trial	15
	Cohort study	10
	Case-series	0
5. Diagnosis certainty (use of preoperative ultrasound, magnetic resonance imaging or post-operative histopathology to confirm diagnosis)	In all	5
	In > 80%	3
	In < 80%, or NS or unclear	0
**Part B—Scores may be given for each option in each of the three sections if applicable**		
1. Outcome criteria	Outcome measures clearly defined	2
	Timing of outcome assessment clearly stated (e.g., at best outcome after injection or at follow-up)	2
2. Procedures for assessing outcomes	Subjects recruited (results not taken from surgeons’ files)	3
	Investigator independent of surgeon/injector	3
	Written assessment	3
3. Description of subject selection process	Selection criteria reported and unbiased	3
	Recruitment rate reported: >80%	4
	Recruitment rate reported: <80%	3

**Table 2:** Authors with two or more top-cited articles.

Author	No. of Articles	Institution(s)/Corporation(s)	Rank of Articles	Total No. of Citations
Anitua	7	Biotechnology Institute Corporation Private Practice in Implantology and Oral Rehabilitation	3, 7, 16, 20, 73, 96, 99	2885
Filardo	4	Rizzoli Orthopedic Institute	54, 68, 69, 88	852
Sanchez, Mikel	3	Arthroscopic Surgery Unit—Hospital Vithas Vitoria	59, 65, 82	641
Ehrenfest	3	The Sahlgrenska Academy at University of Gothenburg Chonnam National University University of Geneva	4, 46, 94	1298
Kon	3	Rizzoli Orthopedic Institute	18, 22, 74	864
Mishra	3	Stanford University	39, 48, 95	709
Weibrich	3	Johannes Gutenberg University of Mainz	10, 11, 93	1010
Eppley	2	Indiana University University of Illinois	5, 17	1094
Marx	2	University of Miami School of Medicine	1,2	3134
Murray	2	Harvard University	61, 85	409
Sundman	2	Cornell University	36, 84	476

**Table 3:** Journals in which the top 100 most cited articles were published.

Journal	Country	Impact Factor (2020)	No. of Articles	Total Citations
*American Journal of Sports Medicine*	USA	6.2	12	2739
*Arthroscopy-The Journal of Arthroscopic and Related Surgery*	UK	4.8	6	1515
*Journal of Orthopedic Research*	USA	3.5	5	1462
*Biomaterials*	Netherlands	12.5	5	1558
*Journal of Periodontology*	USA	2.3	4	1042
*Plastic and Reconstructive Surgery*	USA	4.7	4	1654
*Bone*	USA	4.4	3	810
*International Journal of Oral and Maxillofacial Implants*	USA	2.8	3	1228
*Journal of Oral and Maxillofacial Surgery*	USA	2.8	3	1827
*Knee Surgery Sports Traumatology Arthroscopy*	Germany	4.3	3	791
*Tissue Engineering*	USA	2.6 (2019)	3	698
*Clinical Oral Implants Research*	Denmark	6.0	2	507
*Current Pharmaceutical Biotechnology*	United Arab Emirates	2.8	2	352
*International Journal of Oral and Maxillofacial Surgery*	USA	2.8	2	448
*Journal of Bone and Joint Surgery-American Volume*	USA	5.3	2	453
*Journal of Cellular Physiology*	USA	6.4	2	468
*Osteoarthritis and Cartilage*	UK	6.6	2	470
*Acta Orthopaedica*	UK	3.7	1	178
*American Journal of Physical Medicine and Rehabilitation*	USA	2.2	1	211
*BMC Musculoskeletal Disorders*	UK	2.4	1	214
*Cell Proliferation*	China	6.8	1	176
*Clinical and Experimental Rheumatology*	Italy	4.5	1	220
*Clinical Oral Investigations*	Germany	3.6	1	220
*Clinical Orthopedics and Related Research*	USA	4.3	1	362
*Clinics in Sports Medicine*	USA	2.2	1	262
*Current Reviews in Musculoskeletal Medicine*	USA	*Not* *available*	1	320
*Dermatologic Surgery*	USA	3.4	1	179
*Experimental and Molecular Pathology*	USA	3.4	1	198
*Frontiers in Bioscience-Landmark*	USA	4.0	1	336
*Injury-International Journal of the Care of the Injured*	UK	2.6	1	204
*International Journal of Periodontics and Restorative Dentistry*	USA	1.8	1	204
*Jama-Journal of the American Medical Association*	USA	56.3	1	515
*Journal of Biomedical Materials Research Part B-Applied Biomaterials*	USA	3.4	1	208
*Journal of Bone and Joint Surgery-British Volume*	UK	3.3	1	384
*Journal of Cranio-Maxillofacial Surgery*	UK	2.1	1	421
*Journal of Craniofacial Surgery*	USA	1.0	1	222
*Journal of Dental Research*	USA	6.1	1	246
*Journal of Periodontal Research*	Denmark	4.4	1	221
*Journal of Shoulder and Elbow Surgery*	USA	3.0	1	267
*Journal of the American Academy of Orthopedic Surgeons*	USA	3.0	1	239
*Muscles, Ligaments and Tendons Journal*	Italy	0.4	1	263
*Nature Reviews Rheumatology*	USA	20.5	1	223
*Oral Surgery Oral Medicine Oral Pathology Oral Radiology and Endodontics*	USA	1.2 (2006)	1	1879
*Oral Surgery Oral Medicine Oral Pathology Oral Radiology and Endodontology*	USA	1.5 (2011)	1	291
*Ostomy Wound Management*	USA	2.6	1	265
*Rheumatology*	UK	7.6	1	174
*Sports Medicine*	New Zealand	11.1	1	196
*Stem Cell Research and Therapy*	UK	6.8	1	307
*The Yale Journal of Biology and Medicine*	USA	3.0	1	263
*Thrombosis and Haemostasis*	Germany	5.7	1	958
*Tissue Engineering Part C-Methods*	USA	3.1	1	271
*Transfusion and Apheresis Science*	UK	1.8	1	291
*Trends in Biotechnology*	Netherlands	19.5	1	859

**Table 4:** Subjects investigated among top-cited preclinical studies.

Investigated PRP Subjects	No. of Preclinical Studies
*In vitro*	
Cell proliferation test *	13
Tendon and/or ligament gene expression	2
Inflammation	2
Osteoarthritis	3
Quantification of platelets and/or growth factors	11
Extracellular matrix production of periodontal ligament and osteoblasts	1
*In vitro* and *in vivo*	
Cartilage regeneration	1
Hair growth	1
Meniscus defects	1
*In vivo*	
Bone regeneration	5
Cutaneous wound healing	1
Ligament wound	2
Tendon healing	3

* Adipose-derived stem cells, alveolar bone cells; bone marrow cells; chondrocytes; endothelial cells; fibroblast; mesenchymental stem cells, osteoblasts; periodontally related cells; stromal stem cells; tenocytes.

**Table 5:** PRP indications investigated among top-cited clinical studies.

Investigated PRP Indications	No. of Clinical Studies
Bleeding capillary bed of surgical flaps	1
Cutaneous chronic ulcers	3
Degenerative cartilage lesion or Osteoarthritis	10
Distraction osteogenesis	1
Maxillary sinus or mandibulary grafts	5
Tendinopathy	4
Arthroscopic rotator cuff repair	2

**Table 6:** Promising articles based on the number of citations in the first five years from publication.

Rank	Article	No. of Citations in the First 5 Years (Total Citations)	Type	Modified Coleman Methodology Score	Journal Impact Factor (2020)
1	Guo SC, Tao SC, Yin WJ, Qi X, Yuan T, Zhang CQ. Exosomes derived from platelet-rich plasma promote the re-epithelization of chronic cutaneous wounds via activation of YAP in a diabetic rat model. *Theranostics*. 2017;7(1):81–96. Published 2017 Jan 1. doi:10.7150/thno.16803	160 (160)	Preclinical study	NA	11.6
2	Tao SC, Yuan T, Rui BY, Zhu ZZ, Guo SC, Zhang CQ. Exosomes derived from human platelet-rich plasma prevent apoptosis induced by glucocorticoid-associated endoplasmic reticulum stress in rat osteonecrosis of the femoral head via the Akt/Bad/Bcl-2 signal pathway. *Theranostics*. 2017;7(3):733–750. Published 2017 Jan 15. doi:10.7150/thno.17450	126 (127)	Preclinical study	NA	11.6
3	Fernandes G, Yang S. Application of platelet-rich plasma with stem cells in bone and periodontal tissue engineering. *Bone Res*. 2016;4:16036. Published 2016 Dec 13. doi:10.1038/boneres.2016.36	110 (125)	Review	NA	13.6
4	Smith PA. Intra-articular Autologous Conditioned Plasma Injections Provide Safe and Efficacious Treatment for Knee Osteoarthritis: An FDA-Sanctioned, Randomized, Double-blind, Placebo-controlled Clinical Trial. *Am J Sports Med*. 2016;44(4):884–891. doi:10.1177/0363546515624678	108 (139)	Clinical study	26	6.2
5	Martinez-Zapata MJ, Martí-Carvajal AJ, Solà I, et al. Autologous platelet-rich plasma for treating chronic wounds. *Cochrane Database Syst Rev*. 2016;(5):CD006899. Published 2016 May 25. doi:10.1002/14651858.CD006899.pub3	107 (124)	Meta-analysis	NA	9.3
6	Raeissadat SA, Rayegani SM, Hassanabadi H, et al. Knee Osteoarthritis Injection Choices: Platelet- Rich Plasma (PRP) Versus Hyaluronic Acid (A one-year randomized clinical trial). *Clin Med Insights Arthritis Musculoskelet Disord*. 2015;8:1–8. Published 2015 Jan 7. doi:10.4137/CMAMD.S17894	103 (166)	Clinical study	39	0.5
7	Masuki H, Okudera T, Watanebe T, et al. Growth factor and pro-inflammatory cytokine contents in platelet-rich plasma (PRP), plasma rich in growth factors (PRGF), advanced platelet-rich fibrin (A-PRF), and concentrated growth factors (CGF). *Int J Implant Dent*. 2016;2(1):19. doi:10.1186/s40729-016-0052-4	101 (141)	Preclinical study	NA	2.4
8	Boswell SG, Schnabel LV, Mohammed HO, Sundman EA, Minas T, Fortier LA. Increasing platelet concentrations in leukocyte-reduced platelet-rich plasma decrease collagen gene synthesis in tendons. *Am J Sports Med*. 2014;42(1):42–49. doi:10.1177/0363546513507566	87 (119)	Preclinical study	NA	6.2
9	Campbell KA, Saltzman BM, Mascarenhas R, et al. Does Intra-articular Platelet-Rich Plasma Injection Provide Clinically Superior Outcomes Compared with Other Therapies in the Treatment of Knee Osteoarthritis? A Systematic Review of Overlapping Meta-analyses. *Arthroscopy*. 2015;31(11):2213–2221. doi:10.1016/j.arthro.2015.03.041	85 (129)	Meta-analysis	NA	4.8
10	Martin G, Ricucci D, Gibbs JL, Lin LM. Histological findings of revascularized/revitalized immature permanent molar with apical periodontitis using platelet-rich plasma. *J Endod*. 2013;39(1):138–144. doi:10.1016/j.joen.2012.09.015	83 (142)	Clinical study	17	4.2
11	Chang KV, Hung CY, Aliwarga F, Wang TG, Han DS, Chen WS. Comparative effectiveness of platelet-rich plasma injections for treating knee joint cartilage degenerative pathology: a systematic review and meta-analysis. *Arch Phys Med Rehabil*. 2014;95(3):562–575. doi:10.1016/j.apmr.2013.11.006	81 (147)	Meta-analysis	NA	4.0

## Data Availability

Data is contained within the article or Supplementary Material.

## Supplementary Materials

The following supporting information can be downloaded at: https://www.mdpi.com/article/10.3390/bioengineering9100580/s1, Table S1: Characteristics of the top-cited meta-analyses (level of evidence), preclinical studies (study design and characterization of platelet-rich plasma), and clinical studies (study design, level of evidence, Coleman methodology score (CMS) and characterization of platelet-rich plasma); Table S2: Modified Coleman methodology score (CMS) in top-100 and recent clinical studies.

## Appendix A

**Table A1:** List of the 100 most cited articles in platelets-rich plasma use in regenerative medicine.

Rank	Paper	Country (1st Author)	ACY	No. of Citations
1	Marx RE, Carlson ER, Eichstaedt RM, Schimmele SR, Strauss JE, Georgeff KR. Platelet-rich plasma: Growth factor enhancement for bone grafts. *Oral Surg Oral Med Oral Pathol Oral Radiol Endod*. 1998;85(6):638–646. Doi:10.1016/s1079-2104(98)90029-4	USA	75.16	1879
2	Marx RE. Platelet-rich plasma: evidence to support its use. *J Oral Maxillofac Surg*. 2004; 62(4):489–496. Doi:10.1016/j.joms.2003.12.003	USA	66.05	1255
3	Anitua E, Andia I, Ardanza B, Nurden P, Nurden AT. Autologous platelets as a source of proteins for healing and tissue regeneration. *Thromb Haemost*. 2004;91(1):4–15. Doi:10.1160/TH03-07-0440	Spain	50.42	958
4	Dohan Ehrenfest DM, Rasmusson L, Albrektsson T. Classification of platelet concentrates: from pure platelet-rich plasma (P-PRP) to leucocyte- and platelet-rich fibrin (L-PRF). *Trends Biotechnol*. 2009;27(3):158–167. Doi:10.1016/j.tibtech.2008.11.009	Sweden	61.36	859
5	Eppley BL, Woodell JE, Higgins J. Platelet quantification and growth factor analysis from platelet-rich plasma: implications for wound healing. *Plast Reconstr Surg*. 2004;114(6):1502–1508. Doi:10.1097/01.prs.0000138251.07040.51	USA	39.53	751
6	Foster TE, Puskas BL, Mandelbaum BR, Gerhardt MB, Rodeo SA. Platelet-rich plasma: from basic science to clinical applications. *Am J Sports Med*. 2009;37(11):2259–2272. Doi:10.1177/0363546509349921	USA	52.21	731
7	Anitua E. Plasma rich in growth factors: preliminary results of use in the preparation of future sites for implants. *Int J Oral Maxillofac Implants*. 1999;14(4):529–535.	Spain	28.46	683
8	de Vos RJ, Weir A, van Schie HT, et al. Platelet-rich plasma injection for chronic Achilles tendinopathy: a randomized controlled trial. *JAMA*. 2010;303(2):144–149. Doi:10.1001/jama.2009.1986	Netherlands	39.62	515
9	Chen FM, Zhang M, Wu ZF. Toward delivery of multiple growth factors in tissue engineering. *Biomaterials*. 2010;31(24):6279–6308. Doi:10.1016/j.biomaterials.2010.04.053	China	36.38	473
10	Weibrich G, Kleis WK, Hafner G, Hitzler WE. Growth factor levels in platelet-rich plasma and correlations with donor age, sex, and platelet count. *J Craniomaxillofac Surg*. 2002;30(2):97–102. Doi:10.1054/jcms.2002.0285	Germany	20.05	421
11	Weibrich G, Hansen T, Kleis W, Buch R, Hitzler WE. Effect of platelet concentration in platelet-rich plasma on peri-implant bone regeneration. *Bone*. 2004;34(4):665–671. Doi:10.1016/j.bone.2003.12.010	Germany	21.68	412
12	Patel S, Dhillon MS, Aggarwal S, Marwaha N, Jain A. Treatment with platelet-rich plasma is more effective than placebo for knee osteoarthritis: a prospective, double-blind, randomized trial. *Am J Sports Med*. 2013;41(2):356–364. Doi:10.1177/0363546512471299	India	40.00	400
13	Alsousou J, Thompson M, Hulley P, Noble A, Willett K. The biology of platelet-rich plasma and its application in trauma and orthopaedic surgery: a review of the literature. *J Bone Joint Surg Br*. 2009;91(8):987–996. Doi:10.1302/0301-620X.91B8.22546	England	27.43	384
14	Sánchez AR, Sheridan PJ, Kupp LI. Is platelet-rich plasma the perfect enhancement factor? A current review. *Int J Oral Maxillofac Implants*. 2003;18(1):93–103.	USA	18.40	368
15	Fortier LA, Barker JU, Strauss EJ, McCarrel TM, Cole BJ. The role of growth factors in cartilage repair. *Clin Orthop Relat Res*. 2011;469(10):2706–2715. Doi:10.1007/s11999-011-1857-3	USA	30.17	362
16	Anitua E, Andía I, Sanchez M, et al. Autologous preparations rich in growth factors promote proliferation and induce VEGF and HGF production by human tendon cells in culture. *J Orthop Res*. 2005;23(2):281–286. Doi:10.1016/j.orthres.2004.08.015	Spain	19.67	354
17	Eppley BL, Pietrzak WS, Blanton M. Platelet-rich plasma: a review of biology and applications in plastic surgery. *Plast Reconstr Surg*. 2006;118(6):147e-159e. doi:10.1097/01.prs.0000239606.92676.cf	USA	20.18	343
18	Kon E, Buda R, Filardo G, et al. Platelet-rich plasma: intra-articular knee injections produced favorable results on degenerative cartilage lesions. *Knee Surg Sports Traumatol Arthrosc*. 2010;18(4):472–479. Doi:10.1007/s00167-009-0940-8	Italy	25.85	336
19	Nurden AT, Nurden P, Sanchez M, Andia I, Anitua E. Platelets and wound healing. *Front Biosci*. 2008;13:3532–3548. Published 2008 May 1. Doi:10.2741/2947	France	22.40	336
20	Anitua E, Sánchez M, Orive G, Andía I. The potential impact of the preparation rich in growth factors (PRGF) in different medical fields. *Biomaterials*. 2007;28(31):4551–4560. Doi:10.1016/j.biomaterials.2007.06.037	Spain	20.75	332
21	Landesberg R, Roy M, Glickman RS. Quantification of growth factor levels using a simplified method of platelet-rich plasma gel preparation. *J Oral Maxillofac Surg*. 2000;58(3):297–301. Doi:10.1016/s0278-2391(00)90058-2	USA	14.39	331
22	Kon E, Mandelbaum B, Buda R, et al. Platelet-rich plasma intra-articular injection versus hyaluronic acid viscosupplementation as treatments for cartilage pathology: from early degeneration to osteoarthritis. *Arthroscopy*. 2011;27(11):1490–1501. Doi:10.1016/j.arthro.2011.05.011	Italy	27.00	324
23	Sampson S, Gerhardt M, Mandelbaum B. Platelet rich plasma injection grafts for musculoskeletal injuries: a review. *Curr Rev Musculoskelet Med*. 2008;1(3–4):165–174. Doi:10.1007/s12178-008-9032-5	USA	21.33	320
24	El-Sharkawy H, Kantarci A, Deady J, et al. Platelet-rich plasma: growth factors and pro- and anti-inflammatory properties. *J Periodontol*. 2007;78(4):661–669. Doi:10.1902/jop.2007.060302	USA	19.88	318
25	Man D, Plosker H, Winland-Brown JE. The use of autologous platelet-rich plasma (platelet gel) and autologous platelet-poor plasma (fibrin glue) in cosmetic surgery. *Plast Reconstr Surg*. 2001;107(1):229–239. Doi:10.1097/00006534-200101000-00037	USA	14.36	316
26	Castillo TN, Pouliot MA, Kim HJ, Dragoo JL. Comparison of growth factor and platelet concentration from commercial platelet-rich plasma separation systems. *Am J Sports Med*. 2011;39(2):266–271. Doi:10.1177/0363546510387517	USA	26.17	314
27	Lucarelli E, Beccheroni A, Donati D, et al. Platelet-derived growth factors enhance proliferation of human stromal stem cells. *Biomaterials*. 2003;24(18):3095–3100. Doi:10.1016/s0142-9612(03)00114-5	Italy	15.50	310
28	Amable POur, Carias RB, Teixeira MV, et al. Platelet-rich plasma preparation for regenerative medicine: optimization and quantification of cytokines and growth factors. *Stem Cell Res Ther*. 2013;4(3):67. Published 2013 Jun 7. doi:10.1186/scrt218	Brazil	30.70	307
29	Boswell SG, Cole BJ, Sundman EA, Karas V, Fortier LA. Platelet-rich plasma: a milieu of bioactive factors. *Arthroscopy*. 2012;28(3):429–439. doi:10.1016/j.arthro.2011.10.018	USA	27.82	306
30	Castricini R, Longo UG, De Benedetto M, et al. Platelet-rich plasma augmentation for arthroscopic rotator cuff repair: a randomized controlled trial. *Am J Sports Med*. 2011;39(2):258–265. doi:10.1177/0363546510390780	Italy	25.50	306
31	Graziani F, Ivanovski S, Cei S, Ducci F, Tonetti M, Gabriele M. The in vitro effect of different PRP concentrations on osteoblasts and fibroblasts. *Clin Oral Implants Res*. 2006;17(2):212–219. doi:10.1111/j.1600-0501.2005.01203.x	Italy	17.41	296
32	He L, Lin Y, Hu X, Zhang Y, Wu H. A comparative study of platelet-rich fibrin (PRF) and platelet-rich plasma (PRP) on the effect of proliferation and differentiation of rat osteoblasts in vitro. *Oral Surg Oral Med Oral Pathol Oral Radiol Endod*. 2009;108(5):707–713. doi:10.1016/j.tripleo.2009.06.044	China	20.79	291
33	Crovetti G, Martinelli G, Issi M, et al. Platelet gel for healing cutaneous chronic wounds. *Transfus Apher Sci*. 2004;30(2):145–151. doi:10.1016/j.transci.2004.01.004	Italy	15.32	291
34	DeLong JM, Russell RP, Mazzocca AD. Platelet-rich plasma: the PAW classification system. *Arthroscopy*. 2012;28(7):998–1009. doi:10.1016/j.arthro.2012.04.148	USA	26.27	289
35	Okuda K, Kawase T, Momose M, et al. Platelet-rich plasma contains high levels of platelet-derived growth factor and transforming growth factor-beta and modulates the proliferation of periodontally related cells in vitro. *J Periodontol*. 2003;74(6):849–857. doi:10.1902/jop.2003.74.6.849	Japan	14.45	289
36	Sundman EA, Cole BJ, Fortier LA. Growth factor and catabolic cytokine concentrations are influenced by the cellular composition of platelet-rich plasma. *Am J Sports Med*. 2011;39(10):2135–2140. doi:10.1177/0363546511417792	USA	24.00	288
37	Schnabel LV, Mohammed HO, Miller BJ, et al. Platelet rich plasma (PRP) enhances anabolic gene expression patterns in flexor digitorum superficialis tendons. *J Orthop Res*. 2007;25(2):230–240. doi:10.1002/jor.20278	USA	17.88	286
38	Bendinelli P, Matteucci E, Dogliotti G, et al. Molecular basis of anti-inflammatory action of platelet-rich plasma on human chondrocytes: mechanisms of NF-κB inhibition via HGF. *J Cell Physiol*. 2010;225(3):757–766. doi:10.1002/jcp.22274	Italy	20.85	271
39	Mishra A, Tummala P, King A, et al. Buffered platelet-rich plasma enhances mesenchymal stem cell proliferation and chondrogenic differentiation. *Tissue Eng Part C Methods*. 2009;15(3):431–435. doi:10.1089/ten.tec.2008.0534	USA	19.36	271
40	Mazzocca AD, McCarthy MB, Chowaniec DM, et al. Platelet-rich plasma differs according to preparation method and human variability. *J Bone Joint Surg Am*. 2012;94(4):308–316. doi:10.2106/JBJS.K.00430	USA	24.45	269
41	Randelli P, Arrigoni P, Ragone V, Aliprandi A, Cabitza P. Platelet rich plasma in arthroscopic rotator cuff repair: a prospective RCT study, 2-year follow-up. *J Shoulder Elbow Surg*. 2011;20(4):518–528. doi:10.1016/j.jse.2011.02.008	Italy	22.25	267
42	Akeda K, An HS, Okuma M, et al. Platelet-rich plasma stimulates porcine articular chondrocyte proliferation and matrix biosynthesis. *Osteoarthritis Cartilage*. 2006;14(12):1272–1280. doi:10.1016/j.joca.2006.05.008	USA	15.71	267
43	de Mos M, van der Windt AE, Jahr H, et al. Can platelet-rich plasma enhance tendon repair? A cell culture study. *Am J Sports Med*. 2008;36(6):1171–1178. doi:10.1177/0363546508314430	Netherlands	17.73	266
44	Driver VR, Hanft J, Fylling CP, Beriou JM; Autologel Diabetic Foot Ulcer Study Group. A prospective, randomized, controlled trial of autologous platelet-rich plasma gel for the treatment of diabetic foot ulcers. *Ostomy Wound Manage*. 2006;52(6):.	USA	15.59	265
45	Yamada Y, Ueda M, Naiki T, Takahashi M, Hata K, Nagasaka T. Autogenous injectable bone for regeneration with mesenchymal stem cells and platelet-rich plasma: tissue-engineered bone regeneration. *Tissue Eng*. 2004;10(5–6):955–964. doi:10.1089/1076327041348284	Japan	13.89	264
46	Dohan Ehrenfest DM, Andia I, Zumstein MA, Zhang CQ, Pinto NR, Bielecki T. Classification of platelet concentrates (Platelet-Rich Plasma-PRP, Platelet-Rich Fibrin-PRF) for topical and infiltrative use in orthopedic and sports medicine: current consensus, clinical implications and perspectives. *Muscles Ligaments Tendons J*. 2014;4(1):3–9. Published 2014 May 8.	South Korea Switzerland	29.22	263
47	Lacci KM, Dardik A. Platelet-rich plasma: support for its use in wound healing. *Yale J Biol Med*. 2010;83(1):1–9.	USA	20.23	263
48	Mishra A, Woodall J Jr, Vieira A. Treatment of tendon and muscle using platelet-rich plasma. *Clin Sports Med*. 2009;28(1):113–125. doi:10.1016/j.csm.2008.08.007	USA	18.71	262
49	Schilephake H. Bone growth factors in maxillofacial skeletal reconstruction. *Int J Oral Maxillofac Surg*. 2002;31(5):469–484. doi:10.1054/ijom.2002.0244	Germany	12.48	262
50	Kassolis JD, Rosen PS, Reynolds MA. Alveolar ridge and sinus augmentation utilizing platelet-rich plasma in combination with freeze-dried bone allograft: case series. *J Periodontol*. 2000;71(10):1654–1661. doi:10.1902/jop.2000.71.10.1654	USA	10.96	252
51	Fréchette JP, Martineau I, Gagnon G. Platelet-rich plasmas: growth factor content and roles in wound healing. *J Dent Res*. 2005;84(5):434–439. doi:10.1177/154405910508400507	Canada	13.67	246
52	Kakudo N, Minakata T, Mitsui T, Kushida S, Notodihardjo FZ, Kusumoto K. Proliferation-promoting effect of platelet-rich plasma on human adipose-derived stem cells and human dermal fibroblasts. *Plast Reconstr Surg*. 2008;122(5):1352–1360. doi:10.1097/PRS.0b013e3181882046	Japan	16.27	244
53	Aghaloo TL, Moy PK, Freymiller EG. Investigation of platelet-rich plasma in rabbit cranial defects: A pilot study. *J Oral Maxillofac Surg*. 2002;60(10):1176–1181. doi:10.1053/joms.2002.34994	USA	11.48	241
54	Filardo G, Kon E, Buda R, et al. Platelet-rich plasma intra-articular knee injections for the treatment of degenerative cartilage lesions and osteoarthritis. *Knee Surg Sports Traumatol Arthrosc*. 2011;19(4):528–535. doi:10.1007/s00167-010-1238-6	Italy	20.00	240
55	Niemeyer P, Fechner K, Milz S, et al. Comparison of mesenchymal stem cells from bone marrow and adipose tissue for bone regeneration in a critical size defect of the sheep tibia and the influence of platelet-rich plasma. *Biomaterials*. 2010;31(13):3572–3579. doi:10.1016/j.biomaterials.2010.01.085	Germany	18.46	240
56	McCarrel T, Fortier L. Temporal growth factor release from platelet-rich plasma, trehalose lyophilized platelets, and bone marrow aspirate and their effect on tendon and ligament gene expression. *J Orthop Res*. 2009;27(8):1033–1042. doi:10.1002/jor.20853	USA	17.14	240
57	Hall MP, Band PA, Meislin RJ, Jazrawi LM, Cardone DA. Platelet-rich plasma: current concepts and application in sports medicine [published correction appears in J Am Acad Orthop Surg. 2010 Jan;18(1):17A]. *J Am Acad Orthop Surg*. 2009;17(10):602–608. doi:10.5435/00124635-200910000-00002	USA	17.07	239
58	Ishida K, Kuroda R, Miwa M, et al. The regenerative effects of platelet-rich plasma on meniscal cells in vitro and its in vivo application with biodegradable gelatin hydrogel. *Tissue Eng*. 2007;13(5):1103–1112. doi:10.1089/ten.2006.0193	Japan	14.75	236
59	Sánchez M, Fiz N, Azofra J, et al. A randomized clinical trial evaluating plasma rich in growth factors (PRGF-Endoret) versus hyaluronic acid in the short-term treatment of symptomatic knee osteoarthritis. *Arthroscopy*. 2012;28(8):1070–1078. doi:10.1016/j.arthro.2012.05.011	Spain	20.45	225
60	Andia I, Maffulli N. Platelet-rich plasma for managing pain and inflammation in osteoarthritis. *Nat Rev Rheumatol*. 2013;9(12):721–730. doi:10.1038/nrrheum.2013.141	Spain	22.30	223
61	Murray MM, Spindler KP, Abreu E, et al. Collagen-platelet rich plasma hydrogel enhances primary repair of the porcine anterior cruciate ligament. *J Orthop Res*. 2007;25(1):81–91. doi:10.1002/jor.20282	USA	13.88	222
62	Pietrzak WS, Eppley BL. Platelet rich plasma: biology and new technology. *J Craniofac Surg*. 2005;16(6):1043–1054. doi:10.1097/01.scs.0000186454.07097.bf	USA	12.33	222
63	Camargo PM, Lekovic V, Weinlaender M, Vasilic N, Madzarevic M, Kenney EB. Platelet-rich plasma and bovine porous bone mineral combined with guided tissue regeneration in the treatment of intrabony defects in humans. *J Periodontal Res*. 2002;37(4):300–306. doi:10.1034/j.1600-0765.2002.01001.x	USA	10.52	221
64	Kobayashi E, Flückiger L, Fujioka-Kobayashi M, et al. Comparative release of growth factors from PRP, PRF, and advanced-PRF. *Clin Oral Investig*. 2016;20(9):2353–2360. doi:10.1007/s00784-016-1719-1	Japan Switzerland	31.43	220
65	Sánchez M, Anitua E, Azofra J, Aguirre JJ, Andia I. Intra-articular injection of an autologous preparation rich in growth factors for the treatment of knee OA: a retrospective cohort study. *Clin Exp Rheumatol*. 2008;26(5):910–913.	Spain	14.67	220
66	van Buul GM, Koevoet WL, Kops N, et al. Platelet-rich plasma releasate inhibits inflammatory processes in osteoarthritic chondrocytes. *Am J Sports Med*. 2011;39(11):2362–2370. doi:10.1177/0363546511419278	Netherlands	18.25	219
67	Kitoh H, Kitakoji T, Tsuchiya H, et al. Transplantation of marrow-derived mesenchymal stem cells and platelet-rich plasma during distraction osteogenesis–--a preliminary result of three cases. *Bone*. 2004;35(4):892–898. doi:10.1016/j.bone.2004.06.013	Japan	11.42	217
68	Filardo G, Kon E, Pereira Ruiz MT, et al. Platelet-rich plasma intra-articular injections for cartilage degeneration and osteoarthritis: single- versus double-spinning approach. *Knee Surg Sports Traumatol Arthrosc*. 2012;20(10):2082–2091. doi:10.1007/s00167-011-1837-x	Italy	19.55	215
69	Filardo G, Kon E, Di Martino A, et al. Platelet-rich plasma vs hyaluronic acid to treat knee degenerative pathology: study design and preliminary results of a randomized controlled trial. *BMC Musculoskelet Disord*. 2012;13:229. Published 2012 Nov 23. doi:10.1186/1471-2474-13-229	Italy	19.45	214
70	Spaková T, Rosocha J, Lacko M, Harvanová D, Gharaibeh A. Treatment of knee joint osteoarthritis with autologous platelet-rich plasma in comparison with hyaluronic acid. *Am J Phys Med Rehabil*. 2012;91(5):411–417. doi:10.1097/PHM.0b013e3182aab72	Slovakia	19.18	211
71	Wiltfang J, Kloss FR, Kessler P, et al. Effects of platelet-rich plasma on bone healing in combination with autogenous bone and bone substitutes in critical-size defects. An animal experiment. *Clin Oral Implants Res*. 2004;15(2):187–193. doi:10.1111/j.1600-0501.2004.00980.x	Germany	11.11	211
72	de Jonge S, de Vos RJ, Weir A, et al. One-year follow-up of platelet-rich plasma treatment in chronic Achilles tendinopathy: a double-blind randomized placebo-controlled trial. *Am J Sports Med*. 2011;39(8):1623–1629. doi:10.1177/0363546511404877	Netherlands	17.33	208
73	Anitua E, Aguirre JJ, Algorta J, et al. Effectiveness of autologous preparation rich in growth factors for the treatment of chronic cutaneous ulcers. *J Biomed Mater Res B Appl Biomater*. 2008;84(2):415–421. doi:10.1002/jbm.b.30886	Spain	13.87	208
74	Kon E, Filardo G, Delcogliano M, et al. Platelet-rich plasma: new clinical application: a pilot study for treatment of jumper’s knee. *Injury*. 2009;40(6):598–603. doi:10.1016/j.injury.2008.11.026	Italy	14.57	204
75	Froum SJ, Wallace SS, Tarnow DP, Cho SC. Effect of platelet-rich plasma on bone growth and osseointegration in human maxillary sinus grafts: three bilateral case reports. *Int J Periodontics Restorative Dent*. 2002;22(1):45–53.	USA	9.71	204
76	Zhu Y, Yuan M, Meng HY, et al. Basic science and clinical application of platelet-rich plasma for cartilage defects and osteoarthritis: a review. *Osteoarthritis Cartilage*. 2013;21(11):1627–1637. doi:10.1016/j.joca.2013.07.017	China	20.3	203
77	Xie X, Wang Y, Zhao C, et al. Comparative evaluation of MSCs from bone marrow and adipose tissue seeded in PRP-derived scaffold for cartilage regeneration. *Biomaterials*. 2012;33(29):7008–7018. doi:10.1016/j.biomaterials.2012.06.058	China	18.45	203
78	van den Dolder J, Mooren R, Vloon AP, Stoelinga PJ, Jansen JA. Platelet-rich plasma: quantification of growth factor levels and the effect on growth and differentiation of rat bone marrow cells. *Tissue Eng*. 2006;12(11):3067–3073. doi:10.1089/ten.2006.12.3067	Netherlands	11.65	198
79	Carter CA, Jolly DG, Worden CE Sr, Hendren DG, Kane CJ. Platelet-rich plasma gel promotes differentiation and regeneration during equine wound healing. *Exp Mol Pathol*. 2003;74(3):244–255. doi:10.1016/s0014-4800(03)00017-0	USA	9.90	198
80	Kajikawa Y, Morihara T, Sakamoto H, et al. Platelet-rich plasma enhances the initial mobilization of circulation-derived cells for tendon healing. *J Cell Physiol*. 2008;215(3):837–845. doi:10.1002/jcp.21368	Japan	13.13	197
81	Lopez-Vidriero E, Goulding KA, Simon DA, Sanchez M, Johnson DH. The use of platelet-rich plasma in arthroscopy and sports medicine: optimizing the healing environment. *Arthroscopy*. 2010;26(2):269–278. doi:10.1016/j.arthro.2009.11.015	Canada	15.08	196
82	Sánchez M, Anitua E, Orive G, Mujika I, Andia I. Platelet-rich therapies in the treatment of orthopaedic sport injuries. *Sports Med*. 2009;39(5):345–354. doi:10.2165/00007256-200939050-00002	Spain	14.00	196
83	Krogh TP, Fredberg U, Stengaard-Pedersen K, Christensen R, Jensen P, Ellingsen T. Treatment of lateral epicondylitis with platelet-rich plasma, glucocorticoid, or saline: a randomized, double-blind, placebo-controlled trial. *Am J Sports Med*. 2013;41(3):625–635. doi:10.1177/0363546512472975	Denmark	19.20	192
84	Sundman EA, Cole BJ, Karas V, et al. The anti-inflammatory and matrix restorative mechanisms of platelet-rich plasma in osteoarthritis. *Am J Sports Med*. 2014;42(1):35–41. doi:10.1177/0363546513507766	USA	20.89	188
85	Murray MM, Spindler KP, Ballard P, Welch TP, Zurakowski D, Nanney LB. Enhanced histologic repair in a central wound in the anterior cruciate ligament with a collagen-platelet-rich plasma scaffold. *J Orthop Res*. 2007;25(8):1007–1017. doi:10.1002/jor.20367	USA	11.69	187
86	Choi BH, Zhu SJ, Kim BY, Huh JY, Lee SH, Jung JH. Effect of platelet-rich plasma (PRP) concentration on the viability and proliferation of alveolar bone cells: an in vitro study. *Int J Oral Maxillofac Surg*. 2005;34(4):420–424. doi:10.1016/j.ijom.2004.10.018	Korea	10.33	186
87	Sheth U, Simunovic N, Klein G, et al. Efficacy of autologous platelet-rich plasma use for orthopaedic indications: a meta-analysis. *J Bone Joint Surg Am*. 2012;94(4):298–307. doi:10.2106/JBJS.K.00154	Canada	16.73	184
88	Filardo G, Di Matteo B, Di Martino A, et al. Platelet-Rich Plasma Intra-articular Knee Injections Show No Superiority Versus Viscosupplementation: A Randomized Controlled Trial. *Am J Sports Med*. 2015;43(7):1575–1582. doi:10.1177/0363546515582027	Italy	22.88	183
89	Kawase T, Okuda K, Wolff LF, Yoshie H. Platelet-rich plasma-derived fibrin clot formation stimulates collagen synthesis in periodontal ligament and osteoblastic cells in vitro. *J Periodontol*. 2003;74(6):858–864. doi:10.1902/jop.2003.74.6.858	Japan	9.15	183
90	Schmidmaier G, Herrmann S, Green J, et al. Quantitative assessment of growth factors in reaming aspirate, iliac crest, and platelet preparation. *Bone*. 2006;39(5):1156–1163. doi:10.1016/j.bone.2006.05.023	Germany	10.65	181
91	Li ZJ, Choi HI, Choi DK, et al. Autologous platelet-rich plasma: a potential therapeutic tool for promoting hair growth. *Dermatol Surg*. 2012;38(7 Pt 1):1040–1046. doi:10.1111/j.1524-4725.2012.02394.x	Korea	16.27	179
92	Virchenko O, Aspenberg P. How can one platelet injection after tendon injury lead to a stronger tendon after 4 weeks? Interplay between early regeneration and mechanical stimulation. *Acta Orthop*. 2006;77(5):806–812. doi:10.1080/17453670610013033	Sweden	10.47	178
93	Weibrich G, Kleis WK, Hafner G. Growth factor levels in the platelet-rich plasma produced by 2 different methods: curasan-type PRP kit versus PCCS PRP system. *Int J Oral Maxillofac Implants*. 2002;17(2):184–190.	Germany	8.43	177
94	Dohan Ehrenfest DM, Bielecki T, Jimbo R, et al. Do the fibrin architecture and leukocyte content influence the growth factor release of platelet concentrates? An evidence-based answer comparing a pure platelet-rich plasma (P-PRP) gel and a leukocyte- and platelet-rich fibrin (L-PRF). *Curr Pharm Biotechnol*. 2012;13(7):1145–1152. doi:10.2174/138920112800624382	South Korea France	16.00	176
95	Mishra A, Harmon K, Woodall J, Vieira A. Sports medicine applications of platelet rich plasma. *Curr Pharm Biotechnol*. 2012;13(7):1185–1195. doi:10.2174/138920112800624283	USA	16.00	176
96	Anitua E, Sánchez M, Zalduendo MM, et al. Fibroblastic response to treatment with different preparations rich in growth factors. *Cell Prolif*. 2009;42(2):162–170. doi:10.1111/j.1365–2184.2009.00583.x	Spain	12.57	176
97	Dai WL, Zhou AG, Zhang H, Zhang J. Efficacy of Platelet-Rich Plasma in the Treatment of Knee Osteoarthritis: A Meta-analysis of Randomized Controlled Trials. *Arthroscopy*. 2017;33(3):659–670.e1. doi:10.1016/j.arthro.2016.09.024	China	29.17	175
98	Riboh JC, Saltzman BM, Yanke AB, Fortier L, Cole BJ. Effect of Leukocyte Concentration on the Efficacy of Platelet-Rich Plasma in the Treatment of Knee Osteoarthritis. *Am J Sports Med*. 2016;44(3):792–800. doi:10.1177/0363546515580787	USA	25.00	175
99	Anitua E, Sánchez M, Nurden AT, et al. Platelet-released growth factors enhance the secretion of hyaluronic acid and induce hepatocyte growth factor production by synovial fibroblasts from arthritic patients. *Rheumatology* (Oxford). 2007;46(12):1769–1772. doi:10.1093/rheumatology/kem234	Spain	10.88	174
100	Bosch G, van Schie HT, de Groot MW, et al. Effects of platelet-rich plasma on the quality of repair of mechanically induced core lesions in equine superficial digital flexor tendons: A placebo-controlled experimental study. *J Orthop Res*. 2010;28(2):211–217. doi:10.1002/jor.20980	Netherlands	13.31	173

**Table A2:** Topics and conclusions of the overall top 10 cited articles.

Rank	No. of Citations	ACY	Publication Year	Paper	First Author	Article Type	Theme	Topics and Conclusions
1	1879	75	1998	Platelet-rich plasma-Growth factor enhancement for bone grafts	Marx	Clinical study	Dentistry/ Maxillofacial surgery	Randomized Controlled Trial demonstrating that PRP additions to cancellous cellular bone graft reconstructions of mandibular continuity defects enhanced radiographic maturation and bone density.
2	1255	66	2004	Platelet-rich plasma: Evidence to support its use	Marx	Review	Dentistry/ Maxillofacial surgery	Review about the mechanism of action, platelet dose, therapeutical indications and safety of PRP.
4	958	50	2004	Autologous platelets as a source of proteins for healing and tissue regeneration	Anitua	Review	Multiple	Review about the effects of autologous platelets in different clinical situations requiring accelerated healing and tissue regeneration.
3	859	61	2009	Classification of platelet concentrates: from pure platelet-rich plasma (P-PRP) to leucocyte- and platelet-rich fibrin (L-PRF)	Ehrenfest	Classification	Biology	The review aimed to clarify the different medical devices for the production of PRP and to categorize them under 3 parameters: fibrin density, leucocyte dose and degree of standardization of the procedure.
5	751	40	2004	Platelet quantification and growth factor analysis from platelet-rich plasma: Implications for wound healing	Eppley	Preclinical study	Cosmetic/Plastic sur-gery	Results from this preclinical study demonstrated that platelets can be separated and concentrated 8-fold from whole blood without activating the platelets and providing increased levels of different growth factors, particularly for PDGF-BB, TGF-β1, VEGF and EGF.
6	731	52	2009	Platelet-Rich Plasma From Basic Science to Clinical Applications	Foster	Review	Musculoskeletal system/ Orthopaedics	This article examines the basic science of PRP, and evaluates the human studies that have been published in the ortho-paedic sur-gery and sports medi-cine litera-ture. The use of PRP in amateur and professional sports is reviewed, and the regulation of PRP by anti-doping agen-cies is dis-cussed.
7	683	28	1999	Plasma rich in growth factors: Preliminary results of use in the preparation of future sites for implants	Anitua	Clinical study	Dentistry/Maxillofacial surgery	This clinical study compared epithelialization of areas treated or not with plasma rich in growth factors after dental extraction. Wound healing and bone formation was better in PRGF group.
8	515	40	2010	Platelet-Rich Plasma Injection for Chronic Achilles Tendinopathy A Randomized Controlled Trial	de Vos	Clinical study	Musculoskeletal system/ Orthopaedics	This randomized controlled trial compared eccentric exercises with either a PRP injection or saline injection among patients with chronic Achilles tendinopathy. No benefit on pain and function was observed in PRP group.
9	473	36	2010	Toward delivery of multiple growth factors in tissue engineering	Chen	Review	Multiple	This article summarizes the concept of delivery of multiple growth factors, as well as current efforts to develop sophisticated delivery platforms that allow for controlled spatial presentation and release kinetics of key biological cues.
10	421	20	2002	Growth factor levels in platelet-rich plasma and correlations with donor age, sex, and platelet count	Weibrich	Preclinical study	Biology	Major-age and gender-specific differences in individual growth factor levels were not found. Prediction of resulting growth factor levels based on the thrombocyte counts of whole blood or PRP are not reliable.
