# Supplementary material for: The Top 100 Most Cited Articles on Platelet-Rich Plasma Use in Regenerative Medicine—A Bibliometric Analysis—From the ESSKA Orthobiologic Initiative
Source: Bioengineering (Basel). 2022 Oct 19;9(10):580. doi: 10.3390/bioengineering9100580 (PMC9598782; doi:10.3390/bioengineering9100580)
Supplement: Supplementary file 1 [file bioengineering-09-00580-s001.zip › Supplementary Table 1.pdf]

Table S1. Characteristics of the top-cited meta-analyses (level of evidence), preclinical studies (study design and characterization of platelet-rich plasma) and clinical studies (study design, level of evidence, Coleman methodology score (CMS) and characterization of platelet-rich plasma).

**Rank** *(according to the total number of citations)*	**Paper**	**Article type**	**Study Design**	**Level of Evidence**	**CMS**	**Characteri-zation of PRP**	**Parameters**			
							**PLT**	**WBC**	**RBC**	**≥ 1 GF**
1	Marx RE, Carlson ER, Eichstaedt RM, Schimmele SR, Strauss JE, Georgeff KR. Platelet-rich plasma: Growth factor enhancement for bone grafts. *Oral Surg Oral Med Oral Pathol Oral Radiol Endod*. 1998;85(6):638-646. doi:10.1016/s1079-2104(98)90029-4	Clinical study	Randomized controlled trial	2	39	Incomplete	Yes	No	No	Yes
5	Eppley BL, Woodell JE, Higgins J. Platelet quantification and growth factor analysis from platelet-rich plasma: implications for wound healing. *Plast Reconstr Surg*. 2004;114(6):1502-1508. doi:10.1097/01.prs.0000138251.07040.51	Preclinical study	*In vitro*	NA	NA	Complete	Yes	Yes	Yes	Yes
7	Anitua E. Plasma rich in growth factors: preliminary results of use in the preparation of future sites for implants. *Int J Oral Maxillofac Implants*. 1999;14(4):529-535.	Clinical study	Randomized controlled trial	2	31	Incomplete	Yes	No	No	No
8	de Vos RJ, Weir A, van Schie HT, et al. Platelet-rich plasma injection for chronic Achilles tendinopathy: a randomized controlled trial. *JAMA*. 2010;303(2):144-149. doi:10.1001/jama.2009.1986	Clinical study	Randomized controlled trial	2	27	Absence	No	No	No	No
10	Weibrich G, Kleis WK, Hafner G, Hitzler WE. Growth factor levels in platelet-rich plasma and correlations with donor age, sex, and platelet count. *J Craniomaxillofac Surg*. 2002;30(2):97-102. doi:10.1054/jcms.2002.0285	Preclinical study	*In vitro*	NA	NA	Incomplete	Yes	No	No	Yes
11	Weibrich G, Hansen T, Kleis W, Buch R, Hitzler WE. Effect of platelet concentration in platelet-rich plasma on peri-implant bone regeneration. *Bone*. 2004;34(4):665-671. doi:10.1016/j.bone.2003.12.010	Preclinical study	*In vivo*	NA	NA	Incomplete	Yes	No	No	No
12	Patel S, Dhillon MS, Aggarwal S, Marwaha N, Jain A. Treatment with platelet-rich plasma is more effective than placebo for knee osteoarthritis: a prospective, double-blind, randomized trial. *Am J Sports Med*. 2013;41(2):356-364. doi:10.1177/0363546512471299	Clinical study	Randomized controlled trial	1	37	Incomplete	Yes	Yes	No	No
16	Anitua E, Andía I, Sanchez M, et al. Autologous preparations rich in growth factors promote proliferation and induce VEGF and HGF production by human tendon cells in	Preclinical study	*In vitro*	NA	NA	Incomplete	Yes	No	No	Yes

	culture. *J Orthop Res*. 2005;23(2):281-286. doi:10.1016/j.orthres.2004.08.015									
18	Kon E, Buda R, Filardo G, et al. Platelet-rich plasma: intra-articular knee injections produced favorable results on degenerative cartilage lesions. *Knee Surg Sports Traumatol Arthrosc*. 2010;18(4):472-479. doi:10.1007/s00167-009-0940-8	Clinical study	Case-series	4	24	Incomplete	Yes	No	No	No
21	Landesberg R, Roy M, Glickman RS. Quantification of growth factor levels using a simplified method of platelet-rich plasma gel preparation. *J Oral Maxillofac Surg*. 2000;58(3):297-301. doi:10.1016/s0278-2391(00)90058-2	Preclinical study	*In vitro*	NA	NA	Incomplete	Yes	No	No	No
22	Kon E, Mandelbaum B, Buda R, et al. Platelet-rich plasma intra-articular injection versus hyaluronic acid viscosupplementation as treatments for cartilage pathology: from early degeneration to osteoarthritis. *Arthroscopy*. 2011;27(11):1490-1501. doi:10.1016/j.arthro.2011.05.011	Clinical study	Non-randomized controlled cohort	3	32	Incomplete	Yes	No	No	No
24	El-Sharkawy H, Kantarci A, Deady J, et al. Platelet-rich plasma: growth factors and pro- and anti-inflammatory properties. *J Periodontol*. 2007;78(4):661-669. doi:10.1902/jop.2007.060302	Preclinical study	*In vitro*	NA	NA	Incomplete	Yes	No	No	Yes
25	Man D, Plosker H, Winland-Brown JE. The use of autologous platelet-rich plasma (platelet gel) and autologous platelet-poor plasma (fibrin glue) in cosmetic surgery. *Plast Reconstr Surg*. 2001;107(1):229-239. doi:10.1097/00006534-200101000-00037	Clinical study	Case-series	4	9	Absence	No	No	No	No
26	Castillo TN, Pouliot MA, Kim HJ, Dragoo JL. Comparison of growth factor and platelet concentration from commercial platelet-rich plasma separation systems. *Am J Sports Med*. 2011;39(2):266-271. doi:10.1177/0363546510387517	Preclinical study	*In vitro*	NA	NA	Complete	Yes	Yes	Yes	Yes
27	Lucarelli E, Beccheroni A, Donati D, et al. Platelet-derived growth factors enhance proliferation of human stromal stem cells. *Biomaterials*. 2003;24(18):3095-3100. doi:10.1016/s0142-9612(03)00114-5	Preclinical study	*In vitro*	NA	NA	Incomplete	Yes	No	No	No
28	Amable PR, Carias RB, Teixeira MV, et al. Platelet-rich plasma preparation for regenerative medicine: optimization and quantification of cytokines and growth factors. *Stem Cell Res Ther*. 2013;4(3):67. Published 2013 Jun 7. doi:10.1186/scrt218	Preclinical study	*In vitro*	NA	NA	Complete	Yes	Yes	Yes	Yes

30	Castricini R, Longo UG, De Benedetto M, et al. Platelet-rich plasma augmentation for arthroscopic rotator cuff repair: a randomized controlled trial. *Am J Sports Med*. 2011;39(2):258-265. doi:10.1177/0363546510390780	Clinical study	Randomized controlled trial	1	32	Absence	No	No	No	No
31	Graziani F, Ivanovski S, Cei S, Ducci F, Tonetti M, Gabriele M. The in vitro effect of different PRP concentrations on osteoblasts and fibroblasts. *Clin Oral Implants Res*. 2006;17(2):212-219. doi:10.1111/j.1600-0501.2005.01203.x	Preclinical study	*In vitro*	NA	NA	Incomplete	Yes	No	No	No
32	He L, Lin Y, Hu X, Zhang Y, Wu H. A comparative study of platelet-rich fibrin (PRF) and platelet-rich plasma (PRP) on the effect of proliferation and differentiation of rat osteoblasts in vitro. *Oral Surg Oral Med Oral Pathol Oral Radiol Endod*. 2009;108(5):707-713. doi:10.1016/j.tripleo.2009.06.044	Preclinical study	*In vitro*	NA	NA	Incomplete	Yes	No	No	Yes
33	Crovetti G, Martinelli G, Issi M, et al. Platelet gel for healing cutaneous chronic wounds. *Transfus Apher Sci*. 2004;30(2):145-151. doi:10.1016/j.transci.2004.01.004	Clinical study	Case-series	4	16	Incomplete	Yes	Yes	No	No
35	Okuda K, Kawase T, Momose M, et al. Platelet-rich plasma contains high levels of platelet-derived growth factor and transforming growth factor-beta and modulates the proliferation of periodontally related cells in vitro. *J Periodontol*. 2003;74(6):849-857. doi:10.1902/jop.2003.74.6.849	Preclinical study	*In vitro*	NA	NA	Incomplete	Yes	No	No	Yes
36	Sundman EA, Cole BJ, Fortier LA. Growth factor and catabolic cytokine concentrations are influenced by the cellular composition of platelet-rich plasma. *Am J Sports Med*. 2011;39(10):2135-2140. doi:10.1177/0363546511417792	Preclinical study	*In vitro*	NA	NA	Complete	Yes	Yes	No	Yes
37	Schnabel LV, Mohammed HO, Miller BJ, et al. Platelet rich plasma (PRP) enhances anabolic gene expression patterns in flexor digitorum superficialis tendons. *J Orthop Res*. 2007;25(2):230-240. doi:10.1002/jor.20278	Preclinical study	*In vitro*	NA	NA	Incomplete	Yes	No	No	Yes
38	Bendinelli P, Matteucci E, Dogliotti G, et al. Molecular basis of anti-inflammatory action of platelet-rich plasma on human chondrocytes: mechanisms of NF-κB inhibition via HGF. *J Cell Physiol*. 2010;225(3):757-766. doi:10.1002/jcp.22274	Preclinical study	*In vitro*	NA	NA	Incomplete	Yes	Yes	No	Yes
39	Mishra A, Tummala P, King A, et al. Buffered platelet-rich plasma enhances mesenchymal stem cell proliferation and chondrogenic differentiation. *Tissue Eng Part C Methods*. 2009;15(3):431-435. doi:10.1089/ten.tec.2008.0534	Preclinical study	*In vitro*	NA	NA	Absence	No	No	No	No

40	Mazzocca AD, McCarthy MB, Chowaniec DM, et al. Platelet-rich plasma differs according to preparation method and human variability. *J Bone Joint Surg Am*. 2012;94(4):308-316. doi:10.2106/JBJS.K.00430	Preclinical study	*In vitro*	NA	NA	Complete	Yes	Yes	Yes	Yes
41	Randelli P, Arrigoni P, Ragone V, Aliprandi A, Cabitza P. Platelet rich plasma in arthroscopic rotator cuff repair: a prospective RCT study, 2-year follow-up. *J Shoulder Elbow Surg*. 2011;20(4):518-528. doi:10.1016/j.jse.2011.02.008	Clinical study	Randomized controlled trial	1	26	Absence	No	No	No	No
42	Akeda K, An HS, Okuma M, et al. Platelet-rich plasma stimulates porcine articular chondrocyte proliferation and matrix biosynthesis. *Osteoarthritis Cartilage*. 2006;14(12):1272-1280. doi:10.1016/j.joca.2006.05.008	Preclinical study	*In vitro*	NA	NA	Incomplete	Yes	No	No	Yes
43	de Mos M, van der Windt AE, Jahr H, et al. Can platelet-rich plasma enhance tendon repair? A cell culture study. *Am J Sports Med*. 2008;36(6):1171-1178. doi:10.1177/0363546508314430	Preclinical study	*In vitro*	NA	NA	Complete	Yes	Yes	Yes	Yes
44	Driver VR, Hanft J, Fylling CP, Beriou JM; Autologel Diabetic Foot Ulcer Study Group. A prospective, randomized, controlled trial of autologous platelet-rich plasma gel for the treatment of diabetic foot ulcers. *Ostomy Wound Manage*. 2006;52(6):.	Clinical study	Randomized controlled trial	1	30	Absence	No	No	No	No
45	Yamada Y, Ueda M, Naiki T, Takahashi M, Hata K, Nagasaka T. Autogenous injectable bone for regeneration with mesenchymal stem cells and platelet-rich plasma: tissue-engineered bone regeneration. *Tissue Eng*. 2004;10(5-6):955-964. doi:10.1089/1076327041348284	Preclinical study	*In vivo*	NA	NA	Incomplete	Yes	No	No	No
50	Kassolis JD, Rosen PS, Reynolds MA. Alveolar ridge and sinus augmentation utilizing platelet-rich plasma in combination with freeze-dried bone allograft: case series. *J Periodontol*. 2000;71(10):1654-1661. doi:10.1902/jop.2000.71.10.1654	Clinical study	Case-series	4	14	Incomplete	Yes	No	No	No
51	Fréchette JP, Martineau I, Gagnon G. Platelet-rich plasmas: growth factor content and roles in wound healing. *J Dent Res*. 2005;84(5):434-439. doi:10.1177/154405910508400507	Preclinical study	*In vitro*	NA	NA	Complete	Yes	Yes	Yes	Yes
52	Kakudo N, Minakata T, Mitsui T, Kushida S, Notodihardjo FZ, Kusumoto K. Proliferation-promoting effect of platelet-rich plasma on human adipose-derived stem cells and human dermal fibroblasts. *Plast Reconstr Surg*. 2008;122(5):1352-1360. doi:10.1097/PRS.0b013e3181882046	Preclinical study	*In vitro*	NA	NA	Incomplete	Yes	No	No	Yes

53	Aghaloo TL, Moy PK, Freymiller EG. Investigation of platelet-rich plasma in rabbit cranial defects: A pilot study. *J Oral Maxillofac Surg*. 2002;60(10):1176-1181. doi:10.1053/joms.2002.34994	Preclinical study	*In vivo*	NA	NA	Incomplete	Yes	No	No	No
54	Filardo G, Kon E, Buda R, et al. Platelet-rich plasma intra-articular knee injections for the treatment of degenerative cartilage lesions and osteoarthritis. *Knee Surg Sports Traumatol Arthrosc*. 2011;19(4):528-535. doi:10.1007/s00167-010-1238-6	Clinical study	Case-series	4	24	Incomplete	Yes	No	No	No
55	Niemeyer P, Fechner K, Milz S, et al. Comparison of mesenchymal stem cells from bone marrow and adipose tissue for bone regeneration in a critical size defect of the sheep tibia and the influence of platelet-rich plasma. *Biomaterials*. 2010;31(13):3572-3579. doi:10.1016/j.biomaterials.2010.01.085	Preclinical study	*In vivo*	NA	NA	Incomplete	Yes	Yes	No	Yes
56	McCarrel T, Fortier L. Temporal growth factor release from platelet-rich plasma, trehalose lyophilized platelets, and bone marrow aspirate and their effect on tendon and ligament gene expression. *J Orthop Res*. 2009;27(8):1033-1042. doi:10.1002/jor.20853	Preclinical study	*In vitro*	NA	NA	Incomplete	Yes	Yes	No	Yes
58	Ishida K, Kuroda R, Miwa M, et al. The regenerative effects of platelet-rich plasma on meniscal cells in vitro and its in vivo application with biodegradable gelatin hydrogel. *Tissue Eng*. 2007;13(5):1103-1112. doi:10.1089/ten.2006.0193	Preclinical study	*In vitro and in vivo*	NA	NA	Incomplete	Yes	No	No	Yes
59	Sánchez M, Fiz N, Azofra J, et al. A randomized clinical trial evaluating plasma rich in growth factors (PRGF-Endoret) versus hyaluronic acid in the short-term treatment of symptomatic knee osteoarthritis. *Arthroscopy*. 2012;28(8):1070-1078. doi:10.1016/j.arthro.2012.05.011	Clinical study	Randomized controlled trial	1	30	Absence	No	No	No	No
61	Murray MM, Spindler KP, Abreu E, et al. Collagen-platelet rich plasma hydrogel enhances primary repair of the porcine anterior cruciate ligament. *J Orthop Res*. 2007;25(1):81-91. doi:10.1002/jor.20282	Preclinical study	*In vivo*	NA	NA	Incomplete	Yes	No	No	No
63	Camargo PM, Lekovic V, Weinlaender M, Vasilic N, Madzarevic M, Kenney EB. Platelet-rich plasma and bovine porous bone mineral combined with guided tissue regeneration in the treatment of intrabony defects in humans. *J Periodontal Res*. 2002;37(4):300-306. doi:10.1034/j.1600-0765.2002.01001.x	Clinical study	Split-mouth design	4	15	Absence	No	No	No	No

64	Kobayashi E, Flückiger L, Fujioka-Kobayashi M, et al. Comparative release of growth factors from PRP, PRF, and advanced-PRF. *Clin Oral Investig*. 2016;20(9):2353-2360. doi:10.1007/s00784-016-1719-1	Preclinical study	*In vitro*	NA	NA	Incomplete	No	No	No	Yes
65	Sánchez M, Anitua E, Azofra J, Aguirre JJ, Andia I. Intra-articular injection of an autologous preparation rich in growth factors for the treatment of knee OA: a retrospective cohort study. *Clin Exp Rheumatol*. 2008;26(5):910-913.	Clinical study	Cohort study	3	29	Incomplete	Yes	Yes	No	Yes
66	van Buul GM, Koevoet WL, Kops N, et al. Platelet-rich plasma releasate inhibits inflammatory processes in osteoarthritic chondrocytes. *Am J Sports Med*. 2011;39(11):2362-2370. doi:10.1177/0363546511419278	Preclinical study	*In vitro*	NA	NA	Complete	Yes	Yes	Yes	Yes
67	Kitoh H, Kitakoji T, Tsuchiya H, et al. Transplantation of marrow-derived mesenchymal stem cells and platelet-rich plasma during distraction osteogenesis--a preliminary result of three cases. *Bone*. 2004;35(4):892-898. doi:10.1016/j.bone.2004.06.013	Clinical study	Case-series	4	12	Incomplete	Yes	No	No	No
68	Filardo G, Kon E, Pereira Ruiz MT, et al. Platelet-rich plasma intra-articular injections for cartilage degeneration and osteoarthritis: single- versus double-spinning approach. *Knee Surg Sports Traumatol Arthrosc*. 2012;20(10):2082-2091. doi:10.1007/s00167-011-1837-x	Clinical study	Non-randomized controlled cohort	2	34	Incomplete	Yes	Yes	No	No
69	Filardo G, Kon E, Di Martino A, et al. Platelet-rich plasma vs hyaluronic acid to treat knee degenerative pathology: study design and preliminary results of a randomized controlled trial. *BMC Musculoskelet Disord*. 2012;13:229. Published 2012 Nov 23. doi:10.1186/1471-2474-13-229	Clinical study	Randomized controlled trial	2	39	Incomplete	Yes	Yes	No	No
70	Spaková T, Rosocha J, Lacko M, Harvanová D, Gharaibeh A. Treatment of knee joint osteoarthritis with autologous platelet-rich plasma in comparison with hyaluronic acid. *Am J Phys Med Rehabil*. 2012;91(5):411-417. doi:10.1097/PHM.0b013e3182aab72	Clinical study	Randomized controlled trial	2	37	Incomplete	Yes	Yes	Yes	No
71	Wiltfang J, Kloss FR, Kessler P, et al. Effects of platelet-rich plasma on bone healing in combination with autogenous bone and bone substitutes in critical-size defects. An animal experiment. *Clin Oral Implants Res*. 2004;15(2):187-193. doi:10.1111/j.1600-0501.2004.00980.x	Preclinical study	*In vivo*	NA	NA	Incomplete	Yes	Yes	No	No
72	de Jonge S, de Vos RJ, Weir A, et al. One-year follow-up of platelet-rich plasma treatment in chronic Achilles tendinopathy: a double-blind randomized placebo-controlled	Clinical study	Randomized controlled trial	1	29	Absence	No	No	No	No

	trial. *Am J Sports Med*. 2011;39(8):1623-1629. doi:10.1177/0363546511404877									
73	Anitua E, Aguirre JJ, Algorta J, et al. Effectiveness of autologous preparation rich in growth factors for the treatment of chronic cutaneous ulcers. *J Biomed Mater Res B Appl Biomater*. 2008;84(2):415-421. doi:10.1002/jbm.b.30886	Clinical study	Randomized controlled trial	2	27	Incomplete	Yes	Yes	No	Yes
74	Kon E, Filardo G, Delcogliano M, et al. Platelet-rich plasma: new clinical application: a pilot study for treatment of jumper's knee. *Injury*. 2009;40(6):598-603. doi:10.1016/j.injury.2008.11.026	Clinical study	Non-randomized controlled cohort	3	26	Incomplete	Yes	No	No	No
75	Froum SJ, Wallace SS, Tarnow DP, Cho SC. Effect of platelet-rich plasma on bone growth and osseointegration in human maxillary sinus grafts: three bilateral case reports. *Int J Periodontics Restorative Dent*. 2002;22(1):45-53.	Clinical study	Case-series	4	5	Absence	No	No	No	No
77	Xie X, Wang Y, Zhao C, et al. Comparative evaluation of MSCs from bone marrow and adipose tissue seeded in PRP-derived scaffold for cartilage regeneration. *Biomaterials*. 2012;33(29):7008-7018. doi:10.1016/j.biomaterials.2012.06.058	Preclinical study	*In vitro and in vivo*	NA	NA	Incomplete	Yes	No	No	No
78	van den Dolder J, Mooren R, Vloon AP, Stoelinga PJ, Jansen JA. Platelet-rich plasma: quantification of growth factor levels and the effect on growth and differentiation of rat bone marrow cells. *Tissue Eng*. 2006;12(11):3067-3073. doi:10.1089/ten.2006.12.3067	Preclinical study	*In vitro*	NA	NA	Incomplete	Yes	No	No	Yes
79	Carter CA, Jolly DG, Worden CE Sr, Hendren DG, Kane CJ. Platelet-rich plasma gel promotes differentiation and regeneration during equine wound healing. *Exp Mol Pathol*. 2003;74(3):244-255. doi:10.1016/s0014-4800(03)00017-0	Preclinical study	*In vivo*	NA	NA	Incomplete	Yes	No	No	Yes
80	Kajikawa Y, Morihara T, Sakamoto H, et al. Platelet-rich plasma enhances the initial mobilization of circulation-derived cells for tendon healing. *J Cell Physiol*. 2008;215(3):837-845. doi:10.1002/jcp.21368	Preclinical study	*In vivo*	NA	NA	Incomplete	Yes	No	No	Yes
83	Krogh TP, Fredberg U, Stengaard-Pedersen K, Christensen R, Jensen P, Ellingsen T. Treatment of lateral epicondylitis with platelet-rich plasma, glucocorticoid, or saline: a randomized, double-blind, placebo-controlled trial. *Am J Sports Med*. 2013;41(3):625-635. doi:10.1177/0363546512472975	Clinical study	Randomized controlled trial	1	27	Absence	No	No	No	No

84	Sundman EA, Cole BJ, Karas V, et al. The anti-inflammatory and matrix restorative mechanisms of platelet-rich plasma in osteoarthritis. *Am J Sports Med*. 2014;42(1):35-41. doi:10.1177/0363546513507766	Preclinical study	*In vitro*	NA	NA	Incomplete	Yes	Yes	No	No
85	Murray MM, Spindler KP, Ballard P, Welch TP, Zurakowski D, Nanney LB. Enhanced histologic repair in a central wound in the anterior cruciate ligament with a collagen-platelet-rich plasma scaffold. *J Orthop Res*. 2007;25(8):1007-1017. doi:10.1002/jor.20367	Preclinical study	*In vivo*	NA	NA	Absence	No	No	No	No
86	Choi BH, Zhu SJ, Kim BY, Huh JY, Lee SH, Jung JH. Effect of platelet-rich plasma (PRP) concentration on the viability and proliferation of alveolar bone cells: an in vitro study. *Int J Oral Maxillofac Surg*. 2005;34(4):420-424. doi:10.1016/j.ijom.2004.10.018	Preclinical study	*In vitro*	NA	NA	Incomplete	Yes	No	No	No
87	Sheth U, Simunovic N, Klein G, et al. Efficacy of autologous platelet-rich plasma use for orthopaedic indications: a meta-analysis. *J Bone Joint Surg Am*. 2012;94(4):298-307. doi:10.2106/JBJS.K.00154	Meta-analysis	NA	2	NA	NA	NA	NA	NA	NA
88	Filardo G, Di Matteo B, Di Martino A, et al. Platelet-Rich Plasma Intra-articular Knee Injections Show No Superiority Versus Viscosupplementation: A Randomized Controlled Trial. *Am J Sports Med*. 2015;43(7):1575-1582. doi:10.1177/0363546515582027	Clinical study	Randomized controlled trial	1	39	Incomplete	Yes	Yes	No	No
89	Kawase T, Okuda K, Wolff LF, Yoshie H. Platelet-rich plasma-derived fibrin clot formation stimulates collagen synthesis in periodontal ligament and osteoblastic cells in vitro. *J Periodontol*. 2003;74(6):858-864. doi:10.1902/jop.2003.74.6.858	Preclinical study	*In vitro*	NA	NA	Absence	No	No	No	No
90	Schmidmaier G, Herrmann S, Green J, et al. Quantitative assessment of growth factors in reaming aspirate, iliac crest, and platelet preparation. *Bone*. 2006;39(5):1156-1163. doi:10.1016/j.bone.2006.05.023	Preclinical study	*In vitro*	NA	NA	Incomplete	No	No	No	Yes
91	Li ZJ, Choi HI, Choi DK, et al. Autologous platelet-rich plasma: a potential therapeutic tool for promoting hair growth. *Dermatol Surg*. 2012;38(7 Pt 1):1040-1046. doi:10.1111/j.1524-4725.2012.02394.x	Preclinical study	*In vitro and in vivo*	NA	NA	Incomplete	Yes	No	No	No
92	Virchenko O, Aspenberg P. How can one platelet injection after tendon injury lead to a stronger tendon after 4 weeks? Interplay between early regeneration and mechanical stimulation. *Acta Orthop*. 2006;77(5):806-812. doi:10.1080/17453670610013033	Preclinical study	*In vivo*	NA	NA	Incomplete	Yes	No	No	No

93	Weibrich G, Kleis WK, Hafner G. Growth factor levels in the platelet-rich plasma produced by 2 different methods: curasan-type PRP kit versus PCCS PRP system. *Int J Oral Maxillofac Implants*. 2002;17(2):184-190.	Preclinical study	*In vitro*	NA	NA	Incomplete	Yes	Yes	No	Yes
94	Dohan Ehrenfest DM, Bielecki T, Jimbo R, et al. Do the fibrin architecture and leukocyte content influence the growth factor release of platelet concentrates? An evidence-based answer comparing a pure platelet-rich plasma (P-PRP) gel and a leukocyte- and platelet-rich fibrin (L-PRF). *Curr Pharm Biotechnol*. 2012;13(7):1145-1152. doi:10.2174/138920112800624382	Preclinical study	*In vitro*	NA	NA	Incomplete	No	No	No	Yes
96	Anitua E, Sánchez M, Zalduendo MM, et al. Fibroblastic response to treatment with different preparations rich in growth factors. *Cell Prolif*. 2009;42(2):162-170. doi:10.1111/j.1365-2184.2009.00583.x	Preclinical study	*In vitro*	NA	NA	Incomplete	Yes	Yes	No	Yes
97	Dai WL, Zhou AG, Zhang H, Zhang J. Efficacy of Platelet-Rich Plasma in the Treatment of Knee Osteoarthritis: A Meta-analysis of Randomized Controlled Trials. *Arthroscopy*. 2017;33(3):659-670.e1. doi:10.1016/j.arthro.2016.09.024	Meta-analysis	NA	1	NA	NA	NA	NA	NA	NA
98	Riboh JC, Saltzman BM, Yanke AB, Fortier L, Cole BJ. Effect of Leukocyte Concentration on the Efficacy of Platelet-Rich Plasma in the Treatment of Knee Osteoarthritis. *Am J Sports Med*. 2016;44(3):792-800. doi:10.1177/0363546515580787	Meta-analysis	NA	1	NA	NA	NA	NA	NA	NA
99	Anitua E, Sánchez M, Nurden AT, et al. Platelet-released growth factors enhance the secretion of hyaluronic acid and induce hepatocyte growth factor production by synovial fibroblasts from arthritic patients. *Rheumatology* (Oxford). 2007;46(12):1769-1772. doi:10.1093/rheumatology/kem234	Preclinical study	*In vitro*	NA	NA	Incomplete	Yes	No	No	Yes
100	Bosch G, van Schie HT, de Groot MW, et al. Effects of platelet-rich plasma on the quality of repair of mechanically induced core lesions in equine superficial digital flexor tendons: A placebo-controlled experimental study. *J Orthop Res*. 2010;28(2):211-217. doi:10.1002/jor.20980	Preclinical study	*In vivo*	NA	NA	Incomplete	Yes	Yes	No	Yes

NA: Not Applicable
