# Supplementary material for: The Top 100 Most Cited Articles on Platelet-Rich Plasma Use in Regenerative Medicine—A Bibliometric Analysis—From the ESSKA Orthobiologic Initiative
Source: Bioengineering (Basel). 2022 Oct 19;9(10):580. doi: 10.3390/bioengineering9100580 (PMC9598782; doi:10.3390/bioengineering9100580)
Supplement: Supplementary file 1 [file bioengineering-09-00580-s001.zip › Supplementary Table 2.pdf]

Table S2. Modified Coleman Methodoly Score (CMS) in top-100 and recent clinical studies.

	Title	First author	*Part A - Only one score to be given for each of the sections*					*Part B - Scores may be given for each option in each of the three sections if applicable*								Total Score
								1. Outcome criteria		2. Procedures for assessing outcomes			3. Description of subject selection process			
			Study Size - number of injected site	Mean follow-up (mths)	PRP characterization	Type of study	Diagnosis certainty	Outcome measures clearly defined	Timing of outcome assessment clearly stated	Subjects recruited (results not taken from surgeons' files)	Investigator independent of surgeon /injector	Written assessment	Selection criteria reported and unbiaised	Recruitment rate reported : > 80%	Recruitment rate reported : < 80%	
Top-100	**Platelet-rich plasma - Growth factor enhancement for bone grafts**	**Marx**	10	2	7	15	5	2	2	3	0	0	0	0	0	39
	**Plasma rich in growth factors: Preliminary results of use in the preparation of future sites for implants**	**Anitua**	4	0	7	15	5	0	0	3	0	0	3	0	0	31
	**Platelet-Rich Plasma Injection for Chronic Achilles Tendinopathy A Randomized Controlled Trial**	**De Vos**	7	0	0	15	5	2	2	3	3	3	3	0	0	27
	**Treatment With Platelet-Rich Plasma Is More Effective Than Placebo for Knee Osteoarthritis A Prospective, Double-Blind, Randomized Trial**	**Patel**	10	0	7	15	5	2	2	3	0	3	3	0	0	37
	**Platelet-rich plasma: intra-articular knee injections produced favorable results on degenerative cartilage lesions**	**Kon**	10	2	7	0	5	2	2	3	0	3	3	0	0	24
	**Platelet-Rich Plasma Intra-Articular Injection Versus Hyaluronic Acid Viscosupplementation as Treatments for Cartilage Pathology: From Early Degeneration to Osteoarthritis**	**Kon**	10	0	7	10	5	2	2	3	0	3	3	0	0	32

**The use of autologous platelet-rich plasma (platelet gel) and autologous platelet-poor plasma (fibrin glue) in cosmetic surgery**	**Man**	4	0	0	0	5	0	0	3	0	0	0	0	0	9
**Platelet-Rich Plasma Augmentation for Arthroscopic Rotator Cuff Repair A Randomized Controlled Trial**	**Castricini**	10	2	0	15	5	2	2	3	0	3	3	0	0	32
**Platelet gel for healing cutaneous chronic wounds**	**Crovetti**	4	0	7	0	5	0	2	3	0	0	0	0	0	16
**Platelet rich plasma in arthroscopic rotator cuff repair: a prospective RCT study, 2-year follow-up**	**Randelli**	4	2	0	15	5	2	2	3	3	3	3	0	0	26
**A prospective, randomized, controlled trial of autologous platelet-rich plasma gel for the treatment of diabetic foot ulcers**	**Driver**	10	0	0	15	5	2	2	3	3	0	3	0	3	30
**Alveolar ridge and sinus augmentation utilizing platelet-rich plasma in combination with freeze-dried bone allograft: Case series**	**Kassolis**	0	2	7	0	5	2	2	3	0	0	0	0	0	14
**Platelet-rich plasma intra-articular knee injections for the treatment of degenerative cartilage lesions and osteoarthritis**	**Filardo**	10	2	7	0	5	2	2	3	0	3	3	0	0	24
**A Randomized Clinical Trial Evaluating Plasma Rich in Growth Factors (PRGF-Endoret) Versus Hyaluronic Acid in the Short-Term Treatment of Symptomatic Knee Osteoarthritis**	**Sanchez**	10	0	0	15	5	2	2	3	0	3	3	0	0	30
**Platelet-rich plasma and bovine porous bone mineral combined with guided tissue regeneration in the treatment of intrabony defects in humans**	**Camargo**	0	0	0	10	5	2	2	3	0	0	3	0	0	15

	**Intra-articular injection of an autologous preparation rich in growth factors for the treatment of knee OA: a retrospective cohort study**	**Sanchez**	7	0	7	10	5	2	2	3	0	3	3	0	0	29
	**Transplantation of marrow-derived mesenchymal stem cells and platelet-rich plasma during distraction osteogenesis - a preliminary result of three cases**	**Kitoh**	0	0	7	0	5	0	0	3	0	0	3	0	0	12
	**Platelet-rich plasma intra-articular injections for cartilage degeneration and osteoarthritis: single- versus double-spinning approach**	**Filardo**	10	2	7	10	5	2	2	3	0	3	3	0	0	34
	**Platelet-rich plasma vs hyaluronic acid to treat knee degenerative pathology: study design and preliminary results of a randomized controlled trial**	**Filardo**	10	2	7	15	5	2	2	3	0	3	3	0	0	39
	**Treatment of Knee Joint Osteoarthritis with Autologous Platelet-Rich Plasma in Comparison with Hyaluronic Acid**	**Spakova**	10	0	7	15	5	2	2	3	0	3	3	0	0	37
	**One-Year Follow-up of Platelet-Rich Plasma Treatment in Chronic Achilles Tendinopathy A Double-Blind Randomized Placebo-Controlled Trial**	**de Jonge**	7	2	0	15	5	2	2	3	0	3	3	0	0	29
	**Effectiveness of autologous preparation rich in growth factors for the treatment of chronic cutaneous ulcers**	**Anitua**	0	0	7	15	5	2	2	3	0	0	3	0	0	27
	**Platelet-rich plasma: New clinical application A pilot study for treatment of jumper's knee**	**Kon**	4	0	7	10	5	2	2	3	0	3	3	0	0	26

	**Effect of platelet-rich plasma on bone growth and osseointegration in human maxillary sinus grafts: Three bilateral case reports**	**Froum**	0	0	0	0	5	0	0	3	0	0	3	0	0	5
	**Treatment of Lateral Epicondylitis With Platelet-Rich Plasma, Glucocorticoid, or Saline A Randomized, Double-Blind, Placebo-Controlled Trial**	**Krogh**	7	0	0	15	5	2	2	3	0	3	3	0	0	27
	**Platelet-Rich Plasma Intra-articular Knee Injections Show No Superiority Versus Viscosupplementation: A Randomized Controlled Trial**	**Filardo**	10	2	7	15	5	2	2	3	0	3	3	0	0	39
	**Intra-articular Autologous Conditioned Plasma Injections Provide Safe and Efficacious Treatment for Knee Osteoarthritis: An FDA-Sanctioned, Randomized, Double-blind, Placebo-controlled Clinical Trial**	**Smith**	4	2	0	15	5	2	2	3	3	3	3	0	0	26
**Recent articles**	**Knee Osteoarthritis Injection Choices: Platelet- Rich Plasma (PRP) Versus Hyaluronic Acid (A one-year randomized clinical trial).**	**Raeissad at**	10	2	7	15	5	2	2	3	0	3	3	0	0	39
	**Histological Findings of Revascularized/Revitalized Immature Permanent Molar with Apical Periodontitis Using Platelet-rich Plasma**	**Martin**	0	5	7	0	5	0	0	0	0	0	0	0	0	17
